# Spatiotemporal analysis of RhoA/B/C activation in primary human endothelial cells

**DOI:** 10.1038/srep25502

**Published:** 2016-05-05

**Authors:** Nathalie R. Reinhard, Suzanne F. van Helden, Eloise C. Anthony, Taofei Yin, Yi I. Wu, Joachim Goedhart, Theodorus W. J. Gadella, Peter L. Hordijk

**Affiliations:** 1 University of Amsterdam, Molecular Cytology, Swammerdam Institute for Life Sciences, van leeuwenhoek Centre for Advanced Microscopy, Amsterdam, The Netherlands; 2 Sanquin Research, Molecular Cell Biology, Amsterdam, The Netherlands; 3 Landsteiner Laboratory, Academic Medical Centre, University of Amsterdam, The Netherlands; 4 Center for cell analysis and Modeling, University of Connecticut Health Center, Farmington, United States of America

## Abstract

Endothelial cells line the vasculature and are important for the regulation of blood pressure, vascular permeability, clotting and transendothelial migration of leukocytes and tumor cells. A group of proteins that that control the endothelial barrier function are the RhoGTPases. This study focuses on three homologous (>88%) RhoGTPases: RhoA, RhoB, RhoC of which RhoB and RhoC have been poorly characterized. Using a RhoGTPase mRNA expression analysis we identified RhoC as the highest expressed in primary human endothelial cells. Based on an existing RhoA FRET sensor we developed new RhoB/C FRET sensors to characterize their spatiotemporal activation properties. We found all these RhoGTPase sensors to respond to physiologically relevant agonists (e.g. Thrombin), reaching transient, localized FRET ratio changes up to 200%. These RhoA/B/C FRET sensors show localized GEF and GAP activity and reveal spatial activation differences between RhoA/C and RhoB. Finally, we used these sensors to monitor GEF-specific differential activation of RhoA/B/C. In summary, this study adds high-contrast RhoB/C FRET sensors to the currently available FRET sensor toolkit and uncover new insights in endothelial and RhoGTPase cell biology. This allows us to study activation and signaling by these closely related RhoGTPases with high spatiotemporal resolution in primary human cells.

Endothelial cells (EC) line the vasculature and form a barrier between the blood and the underlying tissue^1^,^2^. Being present in every organ system in the human body, EC control the transport of nutrients and oxygen to tissues and organs, and are the first cells to respond to circulating hormones, metabolites and microvesicle-derived messengers such as microRNAs^3^,^4^. Finally, EC interact, when necessary, with various types of blood cells and platelets in order to orchestrate inflammatory responses and coagulation^5^,^6^,^7^. A key function of EC is the maintenance of the vascular barrier, which limits leakage of plasma or migration of cells into the tissues^8^. It is now well recognized that the endothelium is not a monolayer of passive cells, but actively participates in biological processes central to human health and disease, including the regulation of inflammation, the transendothelial migration (TEM) of various cell types, as well as angiogenesis and arteriogenesis^5^,^9^,^10^,^11^. Many receptor agonists, growth hormones and cytokines regulate the endothelial barrier both positively and negatively^12^, making this a complex feature of human physiology which is essential to understand in detail.

Endothelial permeability to solutes and cells is to a large extent controlled by intercellular contacts. This permeability varies between tissues and for different sections of the same organ^1^,^2^. Inter-endothelial cell-cell contact is commonly determined by two types of junctional complexes, adherens junctions (AJs) and tight junctions (TJs). TJs control the permeability to water, ions and small molecules, and are expressed to a limited extent, in a tissue–specific fashion; i.e. brain endothelium is known for its relatively high numbers of TJs. AJs are usually more abundant in EC contacts and perform more diverse and complex functions in the endothelium^13^,^14^. AJs are formed by Vascular-Endothelial cadherin (VE-cadherin) through homophilic interactions^15^,^16^. VE-cadherin is a calcium-dependent single-span transmembrane adhesion molecule of which the intracellular domain is linked to the actin cytoskeleton via interactions with several adaptor proteins such as α- and β-catenin^17^. Gain and loss of VE-cadherin-mediated adhesion signals towards the actin cytoskeleton thereby controlling endothelial barrier function. This is in part driven by Arp2/3-mediated actin polymerization, which controls lateral membrane protrusions and promotes cell-cell contact, and by acto-myosin-based contractility, which is required for intercellular gap formation^18^.

Inflammatory mediators such as Tumor Necrosis Factor alfa (TNFα) stimulate Nuclear Factor kappaB-mediated expression of leukocyte adhesion receptors (i.e. ICAM-1, VCAM-1) and induce vascular leakage^19^,^20^. A series of interactive, adhesive events between leukocytes and the endothelium allows actin-based morphological changes in both cell types which in turn drive TEM of leukocytes either via the paracellular (through the junctions of adjacent cells) or transcellular route (through the cell body)^5^,^21^,^22^. While this migration of leukocytes serves to eradicate infectious agents and pathogens, excessive TEM is harmful to tissues and the vasculature.

As described above, the endothelium is a dynamic and interactive organ, which for its function and integrity strongly depends on the actin cytoskeleton. A group of proteins that has been actively linked to the regulation of the actin cytoskeleton are the RhoGTPases, guanine nucleotide-binding proteins of approximately 20 kDa^23^,^24^. RhoGTPases are active when bound to GTP and inactive when bound to GDP. In mammals, approximately 20 RhoGTPases have been identified which all show high homology in primary and secondary structure^25^. RhoGTPases are regulated by various groups of proteins, comprising Guanine nucleotide Exchange Factors (GEFs), GTPase Activating Proteins (GAPs) and Guanine nucleotide Dissociation Inhibitors (GDIs)^26^. GEFs promote the exchange of GDP for GTP, activating the RhoGTPase and allowing effector binding and downstream signaling^27^. In turn, GAPs promote GTP hydrolysis, thereby returning RhoGTPases to their inactive, GDP-bound state^26^,^28^,^29^. Finally, GDIs sequester RhoGTPases in the inactive conformation in the cytoplasm, thereby preventing activation, effector binding and proteolytic degradation^30^.

Among the family of RhoGTPases Rac1, Cdc42 and RhoA are the best studied, with Rac1 being linked to Arp2/3-mediated actin polymerization and lamellipodia-driven cell migration, and Cdc42 to filopodia formation^31^,^32^. RhoA is generally accepted to promote myosin activity, actin stress fiber formation and contraction, rather than protrusion^32^. In order to regulate endothelial cytoskeletal dynamics and vascular homeostasis, tight regulation of these RhoGTPases is required. In EC, RhoA-driven contractility is generally accepted to induce a loss of cell-cell contacts and an increase in permeability induced by agonists such as histamine or Thrombin^33^,^34^. Whereas activation of RhoA through these agonists is well described, it is unclear if this response is unique to RhoA or whether, in parallel, related Rho family members such as RhoB or RhoC are activated as well.

Here we addressed this topic by performing a RhoGTPase mRNA expression analysis in primary human EC. Unexpectedly, we found that RhoC is the highest expressed RhoGTPase in primary human EC at the level of mRNA. By using a novel FRET-based biosensor, we characterized RhoC activation in live primary human EC, and include a similar FRET-analyses of its closest homologues RhoA and RhoB. We found that these GTPases are rapidly activated in parallel by Thrombin, but with spatially distinct characteristics. These findings underscore the feasibility of using such closely related GTPases in biosensors to delineate detailed, stimulus-specific spatio-temporal activation in live human EC.

## Results

### RhoC is highly expressed in EC and colocalizes with VE-cadherin

**Figure 1:**
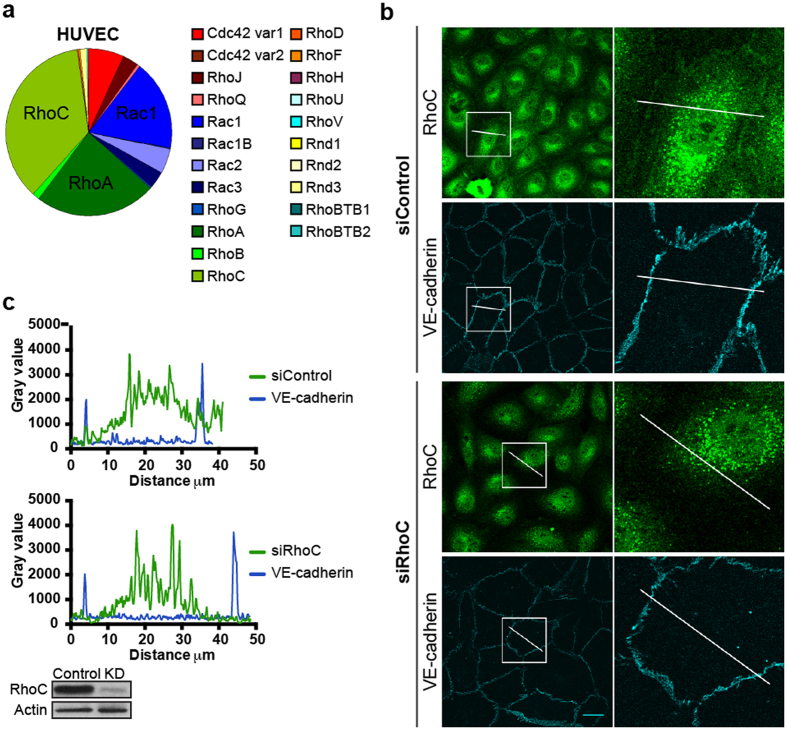
RhoC is highly expressed in human EC and colocalizes with VE-cadherin. (**a**) RhoGTPase qPCR analysis in HUVEC. Pie chart displays mean values of 3 sequential qPCR experiments. (**b**) EC were transfected with siRNA control or siRhoC and stained for RhoC and VE-cadherin. Boxes show co-localization of RhoC and VE-cadherin in Control cells, but not in RhoC knockdown cells. Bar = 25 μm. (**c**) Gray value profile of RhoC (green line) and VE-cadherin (blue line) in siControl and siRhoC EC according to the lines present in the box selections in (**b**). Efficiency of RhoC Knockdown was checked on Western blot, actin was detected to control for equal loading.

Previous studies on RhoGTPases in EC have mainly focused on the well-characterized proteins Rac1, Cdc42 and RhoA. To examine whether additional RhoGTPases may be relevant for EC function, an expression analysis was performed to quantify mRNA expression levels of different RhoGTPases in EC^35^. In this approach, HUVEC mRNA was amplified using well-defined primer sets that were extensively screened for specificity. To correct for differences in the amount of total RNA input and for RT-efficiency, the quantity of the Rho GTPase transcripts was normalized to the amount of β-glucuronidase gene transcripts^35^. The RhoGTPase RhoC (40%) was found to be the highest expressed RhoGTPase in HUVEC, followed by its closest relative RhoA (25%), and Rac1 (20%) and Cdc42 (8%). Lower expression levels were observed for RhoB (2%), the RhoGTPase that shows the highest homology in amino acid sequence and structure to RhoA and RhoC (Fig. 1a).

To explore the most abundant RhoGTPase in more detail, we focused on the localization of endogenous RhoC using immunofluorescence. We first tested an existing RhoC antibody by sequentially overexpressing RhoA- and RhoC-GFP in EC with equal expression levels. Western blot analyses showed endogenous RhoC expression in both samples (Supplemental Fig. S1). Comparing RhoA-GFP and RhoC-GFP, only RhoC-GFP was readily detected, indicating that this antibody is specific for RhoC and does not crossreact with RhoA. RhoC immunostaining revealed a relatively high signal in the cytoplasm and the nucleus, in addition to detectable RhoC at cell-cell contacts (Fig. 1b). To determine which part of this staining was specific, we transfected EC with RhoC-specific siRNA, prior to immunostaining. This data showed that in siRNA RhoC transfected EC, the junctional immunostaining was no longer detectable, suggesting that a fraction of RhoC resides at or near junctions, co-localizing with the endothelial junctional molecule VE-cadherin (Fig. 1b,c). This junctional staining may be the direct result of RhoC plasma membrane association and the concomitant accumulation of signal at sites of overlapping junctional membranes. In addition, there exists a cytoplasmic, diffusely localized pool of RhoC, as deduced from the loss in signal in the cytoplasm due to the siRNA expression.

Together, this initial analysis reveals that, in addition to Rac1, Cdc42 and RhoA, RhoC is abundantly expressed in primary human EC, where it shows specific localization at cell-cell contacts as well as in the cytoplasm.

### Thrombin induces RhoC activation, intercellular gap formation and loss of RhoC from cell-cell contacts

**Figure 2:**
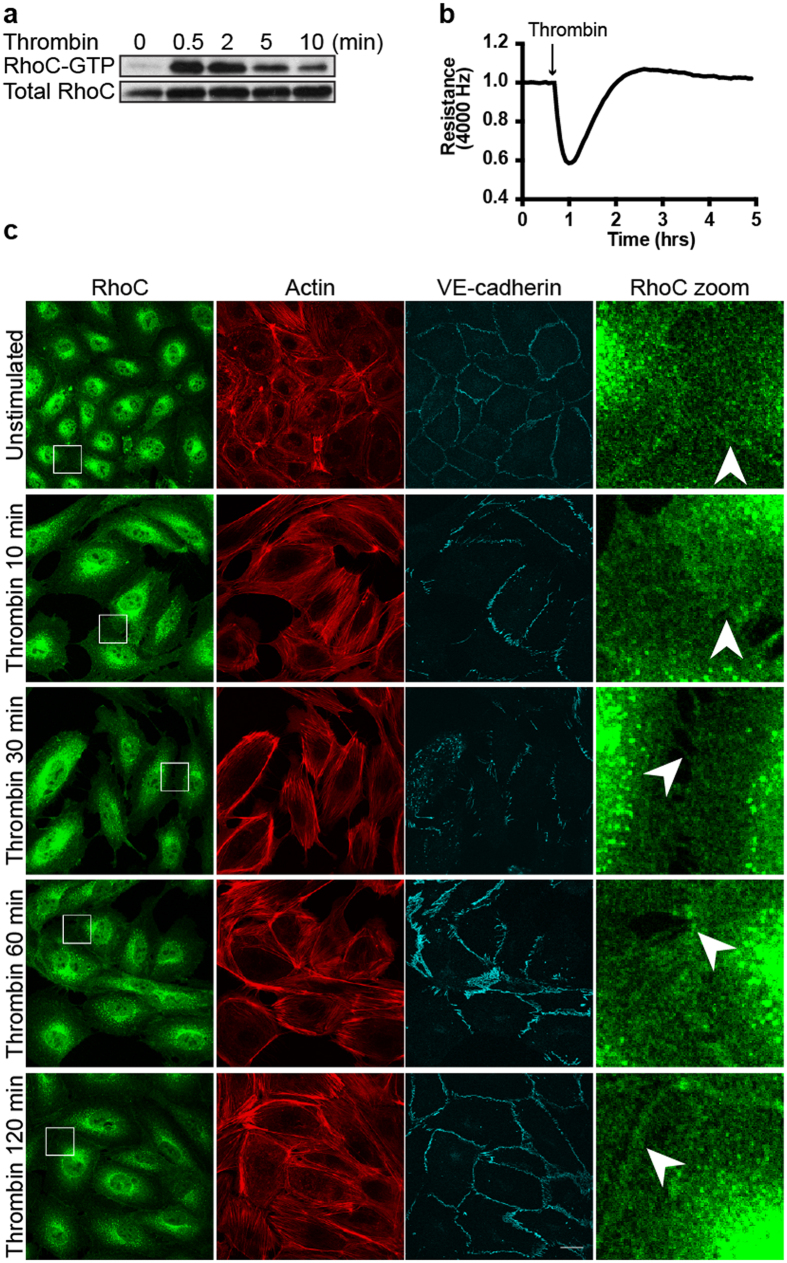
Thrombin induces RhoC activation and a transient loss of RhoC at cell-cell contacts. (**a**) RhoC-GTP levels were increased in EC after Thrombin (1 U/ml) stimulation, as analyzed using a Rhotekin-RBD pull down. Total RhoC levels were detected to control for equal RhoC levels and loading. (**b**) EC were grown to a monolayer on electrode arrays. Resistance of the endothelial monolayer was measured on the ECIS before and after stimulation of thrombin at t = 0:41 hours. (**c**) EC were stained for RhoC, Actin and VE-cadherin and were followed over time after Thrombin stimulation (1 U/ml). Arrowheads indicate RhoC-specific cell-cell contact areas where RhoC is present in unstimulated EC, disappears at Thrombin = 10, 30 min and relocates at Thrombin = 60, 120 min. Bar = 25 μm.

Following up on the analysis of its localization, we characterized RhoC in EC further by detecting its activation using a GST-Rhotekin-based pull-down assay^36^. In these experiments, we tested whether RhoC in primary human EC is activated by the protease Thrombin, a well-established activator of RhoA and a barrier-disrupting agonist for EC^37^. In addition, Thrombin stimulates the release of inflammatory mediators, vasoregulators, and growth factors and induces leukocyte adhesion in the process of TEM^38^. Here we show that a brief (30 sec.) stimulation of EC with Thrombin strongly activates RhoC (Fig. 2a). This activation gradually decreased at 2, 5 and 10 minutes respectively. RhoC activation preceded the Thrombin-induced loss in transendothelial electrical resistance as measured by ECIS (Fig. 2b). To determine if RhoC localization at cell-cell contacts was affected by Thrombin, we detected endogenous RhoC using immunostaining following Thrombin stimulation. Unstimulated, confluent EC contain mainly cortical actin and form stable cell-cell junctions to which RhoC is localized (Fig. 2c). We found that, in addition to the induction of actin stress fibers and intercellular gaps, Thrombin caused a loss of RhoC from cell-cell contacts detected at 10 min after stimulation. After 1–2 hrs of Thrombin treatment, recovery of the endothelial monolayer occurred, together with the reorganization of F-actin and VE-cadherin and the re-localization of RhoC at cell-cell contacts (Fig. 2c).

Collectively, these data show that Thrombin efficiently activates RhoC, resulting in the relocation of this protein away from cell-cell contacts, the induction of cell contraction and endothelial barrier disruption.

### RhoB/C sensor development based on a currently existing RhoA FRET sensor

**Figure 3:**
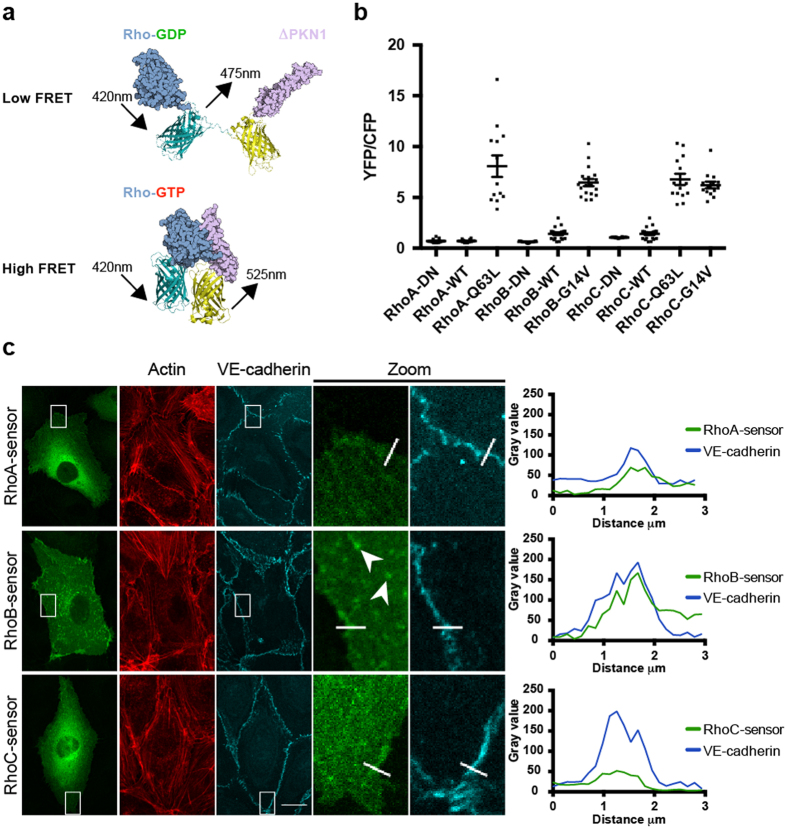
Development of RhoB/C- and localization of RhoA/B/C FRET sensors. (**a**) Intramolecular RhoGTPase FRET sensor design that consists of (from N- to C-terminal) the RBD of PKN1 (ΔPKN), YFP (mVenus), CFP (Cerulean3) and a RhoGTPase. RhoGTPase activation results in RBD-RhoGTP binding and an energy transfer from CFP to YFP. (**b**) Average basal YFP/CFP ratios (±SEM) of RhoA-NB (n = 13), RhoA-WT (n = 16), RhoA-Q63L (n = 13), RhoB-NB (n = 17), RhoB-WT (n = 20), RhoB-G14V (n = 18), RhoC-NB (n = 14), RhoC-WT (n = 20), RhoC-Q63L (n = 14) and RhoC-G14V (n = 14) FRET sensors expressed in EC. (**c**) EC were transfected with RhoA/B/C-WT FRET sensors and stained for actin and VE-cadherin. Boxes show co-localization of the FRET sensors with VE-cadherin, corresponding profile plots show gray values for RhoA/B/C-WT FRET sensors (green line) and VE-cadherin (blue line) according to the lines selected in the box regions. Arrowheads indicate vesicular localization of the RhoB-WT sensor. Bar = 14 μm.

Although the Rhotekin-RBD pull down experiment clearly showed RhoC activation, this biochemical approach is limited due to the lack of high-resolution temporal and spatial information. In order to gain detailed knowledge regarding localized activation of RhoC, we decided to pursue a strategy based on the use of an intramolecular FRET sensor, derived from a previously developed, high-contrast RhoA FRET sensor^39^,^40^. This single-chain biosensor comprises (N-to-C-terminal): (i) a circularly permutated RBD derived from the Rho effector PKN1; (ii) two fluorescent proteins (YFP and CFP derivatives; mVenus and Cerulean3); and (iii) the RhoA GTPase itself. This configuration of the sensor allows proper truncation, carboxymethylation and lipidation of the RhoA C-terminal CAAX box and allows the hypervariable C-terminal domain to mediate functionally relevant protein-protein interactions^41^. Once GDP-GTP exchange on RhoA is stimulated by GEF activity, this sensor design allows RhoA-GTP-PKN1 binding which will promote energy transfer from the CFP to the then closely apposed YFP (Fig. 3a, reproduced from^40^). In other words, YFP/CFP ratios can be used as readout for the nucleotide-binding state of RhoA activation.

Building on this RhoA FRET sensor (detailed validation and characterization will be published separately), we developed FRET sensor variants for its two closest relatives RhoB and RhoC, including the wildtype (WT) GTPase sensors, non-binding (NB) effector mutant sensors and dominant positive GTPase mutant sensors. In the NB RhoB/C sensors, similar to the RhoA-NB sensor, PKN1-WT is replaced by PKN1-L59Q, a non-binding mutant of PKN1. This single amino acid effector mutant prevents binding of activated RhoA/B/C to the PKN1 effector domain. In addition, we also introduced various constitutively active RhoB/C mutants in the FRET constructs. Using locked, GTP-bound variants of RhoB/C, we generated RhoB-G14V-, RhoC-G14V- and RhoC-Q63L FRET sensors (Fig. 3b). Spectral imaging of the DN and dominant positive RhoB/C sensors was performed following expression in HEK293 cells (Supplemental Fig. S2).

We used transient expression of the sensor constructs in primary human EC to analyze basal and stimulated activity and activity distribution in live cells. Comparing different RhoB FRET sensor variants expressed in resting EC, we found the lowest YFP/CFP ratio for RhoB-NB (0.6), followed by RhoB-WT (1.4) and RhoB-G14V (6.5). Comparable results were obtained for the RhoC sensor variants, RhoC-NB (1.1), RhoC-WT (1.4) and RhoC-G14V (6.2), respectively. Moreover, no difference was observed between RhoC-G14V (6.2) and RhoC-Q63L (6.8). In contrast to RhoB/C, RhoA-NB (0.7) and RhoA-WT (0.7) did not differ, whereas RhoA-Q63L (8.1) showed a more than 10-fold increase in YFP/CFP ratio compared to both RhoA-NB and RhoA-WT (Fig. 3b).

To examine the localization of the RhoA/B/C-WT sensors we combined expression of these sensors with immunofluorescence for actin and VE-cadherin. Confocal imaging revealed that both RhoA/C-WT mainly showed cytosolic localization, whereas RhoB was also present in vesicular structures, confirming previous findings showing localization of RhoB to endosomes^42^,^43^. The localization of these FRET sensors were also similar to their corresponding CFP- or GFP-tagged variants (Supplemental Fig. S3). In addition, all three RhoGTPase WT FRET sensors partially co-localized with VE-cadherin, an observation congruent with endogenous RhoC localization (Figs 1b,c and 3c). This data confirms that the localization of the sensors is in line with the localization of the endogenous RhoGTPases. This is an important feature, which we propose is the due to the C-terminus of the GTPases being readily accessibly for post-translational modifications and the binding of regulatory or targeting proteins.

In summary, we constructed new, high-contrast RhoB/C FRET sensors based on an existing RhoA FRET sensor. All dominant active FRET sensor mutants showed higher YFP/CFP ratios (up to >10-fold) as compared to their cognate NB and WT mutants.

### Thrombin induces spatial activation differences between RhoA/C and RhoB

**Figure 4:**
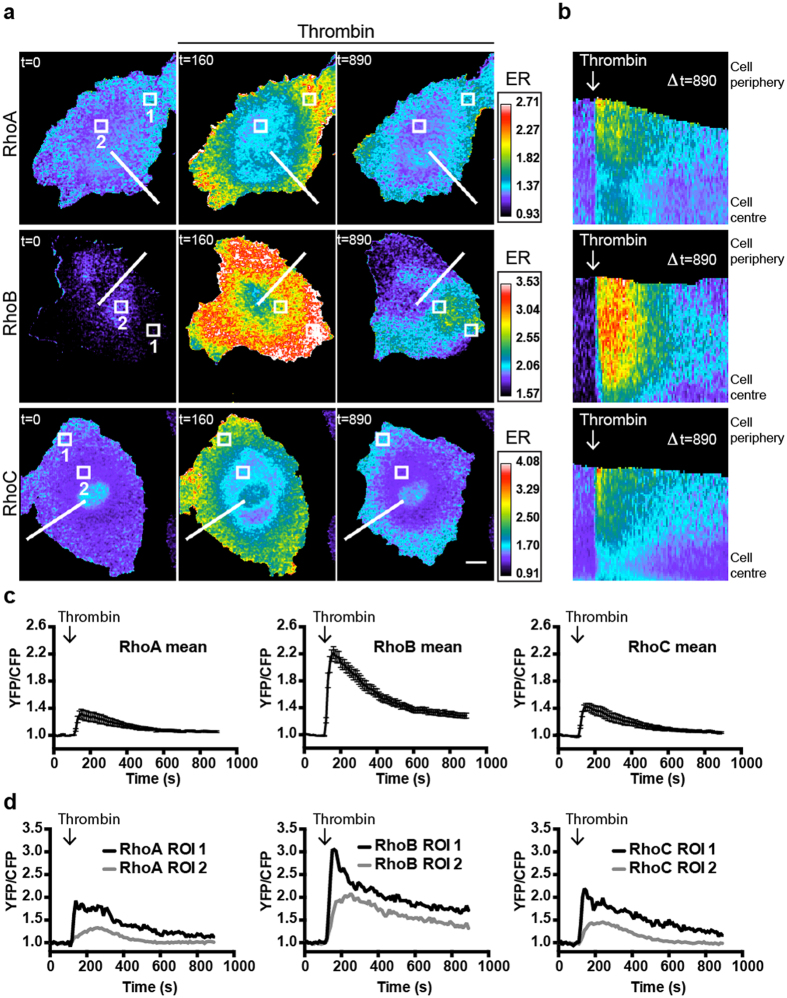
Thrombin induces RhoA/B/C activation in human EC. (**a**) Ratiometric images of EC that were sequentially transfected with RhoA/B/C-WT FRET sensors and stimulated with Thrombin (1 U/ml) at t = 110. Warm colors represent high YFP/CFP ratios (Emission ratio (ER) on the right). Bar = 10 μm, t = in seconds. (**b**) Kymograph analysis of Thrombin stimulated RhoA/B/C-WT FRET sensors corresponding to the lines indicated in (**a**). (**c**) Normalized mean YFP/CFP ratio changes (±SEM) of the RhoA-WT (n = 13), RhoB-WT (n = 22) and RhoC-WT (n = 10) sensors after Thrombin stimulation at t = 110. (**d**) Normalized YFP/CFP ratio changes of localized RhoA/B/C activation in ROIs (Region Of Interest) indicated in (**a**).

Since Thrombin is a known activator of RhoA^37^, we used these novel sensors to analyze Thrombin-induced activation of RhoB/C and compared the activation profiles of the RhoA/B/C-WT FRET sensors. Sensor-expressing EC were stimulated with Thrombin and showed fast sub-second YFP/CFP ratio increases that returned to basal levels between 10 and 15 minutes in all three conditions (Fig. 4a–c, Supplemental Movie 1). These fast FRET sensor activation dynamics are in line with endogenous RhoA/C activation, measured using a biochemical approach (Fig. 2a)^37^. RhoA/C-WT sensors show similar activation properties in terms of mean maximal FRET ratio changes (RhoA 32% at t = 40 sec after Thrombin, RhoC 42% at t = 40 sec after Thrombin) as well as for the localization of the activity. Both RhoA and RhoC showed mainly peripheral activation, in contrast to RhoB activation which was distributed more throughout the cytoplasm and then condensed into intracellular regions of activity before declining to close-to-basal values. Similar spatial FRET ratio differences between RhoA/C and RhoB were also observed for the cognate dominant positive sensor mutants (Supplemental Fig. S4). Notably, higher maximal FRET ratio changes were consistently observed for the RhoB-WT sensor (max 122% at t = 50 sec after Thrombin). These RhoA/B/C FRET ratio changes (Fig. 4c) are calculated for entire, FRET sensor expressing cells, but localized regions within single EC show heterogeneity in terms of maximal FRET ratio change (Fig. 4d). In peripheral high activation regions, we can detect up to a 200% maximal increase in FRET ratio (RhoA 93%, RhoB 205%, RhoC 120%), while in regions towards the cell center FRET ratio differences are less pronounced (RhoA 33%, RhoB 108%, RhoC 46%). Furthermore, we did not observe any correlation between expression level and Thrombin-induced activation, indicating that the FRET signals are sensor concentration-independent.

Together these data reveal robust Thrombin-induced RhoA/B/C-WT FRET sensor activation with high temporal and spatial resolution, with the RhoB sensor showing the largest FRET ratio change. In addition, while RhoA/C activation was mainly peripherally localized, RhoB activation was found to be more homogeneously spread throughout the EC.

### RhoA/B/C sensors can be used as a general Rho activation readout

**Figure 5:**
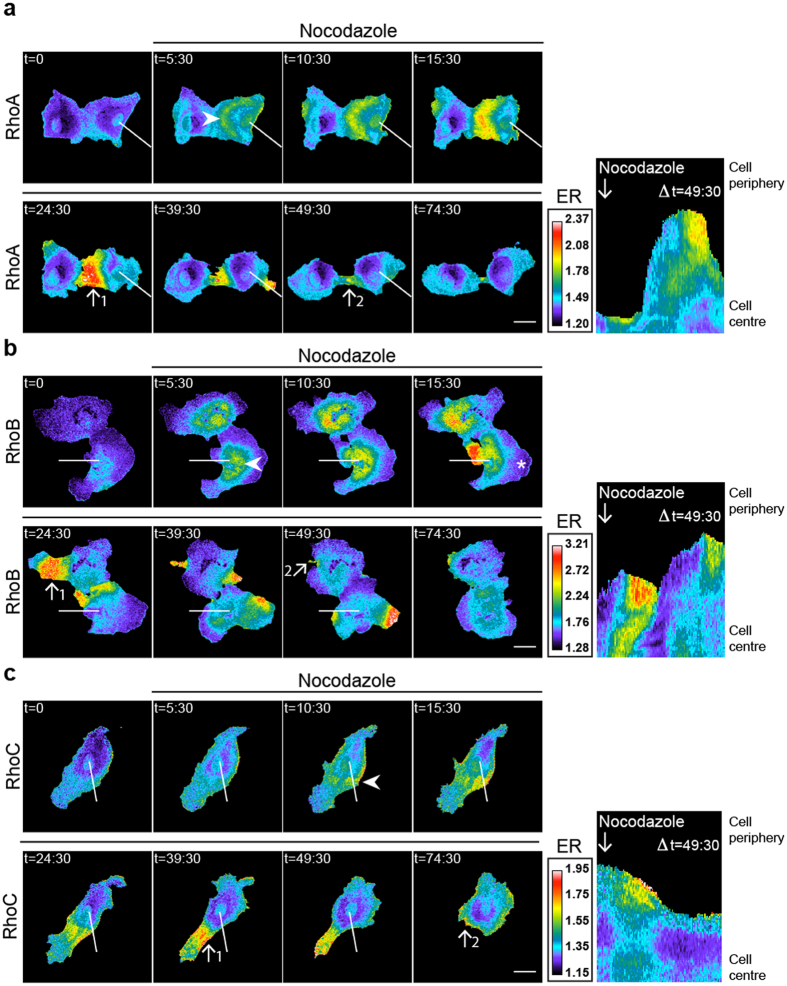
Nocodazole activates RhoA/B/C in spatial activation zones. (**a**–**c**) Ratiometric images of EC that were sequentially transfected with RhoA/B/C-WT FRET sensors and stimulated with 5 μM Nocodazole at t =  3:00, t = in minutes. Warm colors represent high YFP/CFP ratios (Emission ratio (ER) on the right). Kymograph analysis is based on the indicated line and correspond to the time frame of in t = 0–49’:30”. Arrowheads indicate peripheral start activation for RhoA/C in (**a,c**) and perinuclear start activation for RhoB in (**b**). Arrows display pre-contractile, high activation (#1) and post-contractile, low activation (#2) zones in (**a**–**c**). Asterisk shows low RhoB activation in a protrusive area in (**b**). Bar = 25 μm.

The RhoA/B/C-WT sensors responded to the GPCR agonist Thrombin, as well as histamine (Supplemental Figs S5,6,7), while no FRET changes were observed upon 0–15 min. stimulation of VEGF and TGFβ respectively (Supplemental Figs S5,6,7). To determine whether additional, established Rho-activating mechanisms can activate these sensors, EC were stimulated with nocodazole, a compound that inhibits microtubule (MT) polymerization, the result of which is the release and activation of MT-associated GEFH1^44^. GEFH1 activates RhoA, leading to the formation of F-actin stress fibers^44^,^45^. Nocodazole treatment of sensor-expressing EC results in activation of all three RhoGTPases (Fig. 5a–c, Supplemental Movie S2). Interestingly, the RhoA/B/C activation induced by nocodazole was long-lasting and more spatially restricted as compared to the Thrombin responses (compare Figs 4a and 5a–c, Supplemental Movie S2). Activation of RhoB was initially detected as a field of increased FRET ratio within the cell body, after which regions of RhoB activation translocated through the cell body in a seemingly cyclic fashion. The RhoB activation zone occurred in protruded areas, consistently preceeding cellular contraction, while protrusions, devoid of such activation, were visible on the other side of the cell. The activation zone next translocated towards protrusive areas, to be followed by local contraction (also visualized by kymograph analysis in Fig. 5b). These persistent, motile zones could be detected for the duration of the movies, and could last for over at least 75 minutes. In contrast to RhoB, RhoA/C activation started at the cell periphery. For all three GTPases, we found that formation of these localized zones of high activation preceded cell contraction (Fig. 5a–c).

In summary, we observed RhoA/B/C activation in EC upon stimulation with the non-GPCR specific stimulus nocodazole. Activation patterns of these three proteins were highly localized and dynamic, with initial RhoB activation detected in a large perinuclear area, whereas initial RhoA/C activation was consistently peripheral.

### The pro-inflammatory cytokine TNFα upregulates and activates RhoB

In the above-described experiments we used stimuli that can activate RhoA, RhoB and RhoC. To investigate if the RhoGTPase FRET sensors can be used as readout for specific RhoGTPase activation, we used TNFα, a cytokine that predominantly activates RhoB, as was shown biochemically^46^. Compared to RhoA/C, basal levels of total RhoB protein are hardly detectable by Western Blot. However, in contrast to RhoA, RhoB is strongly upregulated in human EC upon stimulation with TNFα^46^. Whether these increased protein levels correspond to increased RhoB activation has not been previously addressed.

**Figure 6:**
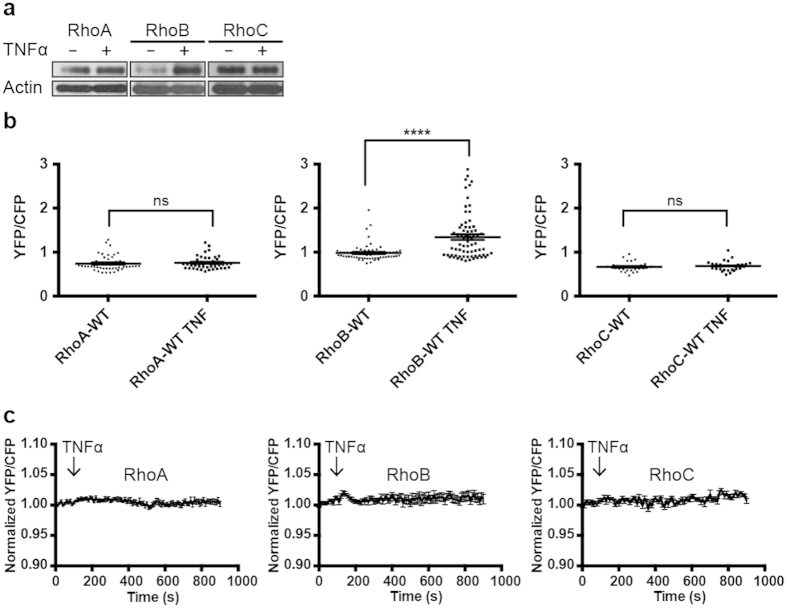
TNFα-induced upregulation and activation of RhoB. (**a**) Untreated and TNFα-treated (10 ng/ml, 16 hours) EC were lysed and protein levels of RhoA/B/C were detected on Western blot. Actin was detected to control for equal loading. (**b**) EC were transfected with the RhoA/B/C FRET sensors as indicated. YFP/CFP ratios (±SEM) were compared for untreated RhoA (n = 47), RhoB (n = 65), RhoC (n = 30) and TNFα-treated (10 ng/ml, 16 hours) RhoA (n = 41), RhoB (n = 70), RhoC (n = 28) sensor-expressing EC, ns = P > 0.05, ****P<0.0001, Student’s t test. (**c**) Normalized mean YFP/CFP ratios (±SEM) of EC that were transfected with the RhoA (n = 11), RhoB (n = 13) or RhoC (n = 12) FRET sensor and stimulated with TNFα at t = 110 sec.

By stimulating EC with TNFα for 16 hours, we were able to reproduce the finding^46^ that total protein levels for RhoA remained unaffected whereas RhoB was strongly upregulated (Fig. 6a). Furthermore, including RhoC in this experiment showed that TNFα did not alter RhoC GTPase expression levels. To test if the increased RhoB protein level is equivalent to an increase in RhoB activation, we applied the same TNFα treatment for 16 hrs to RhoA/B/C-WT sensor expressing EC. Comparing untreated and TNFα-treated cells revealed significant higher YFP/CFP ratios for the RhoB FRET sensor upon TNFα stimulation (p < 0.0001), indicative for increased RhoB activation (Fig. 6b). This TNFα-induced RhoB activation was induced relatively late, since no change in YFP/CFP ratios was observed within the first 15 minutes after TNFα stimulation (Fig. 6c). In contrast to the findings with RhoB, RhoA (p = 0.63) and RhoC (p = 0.54) FRET ratios remained stable during both the long- and the short-term TNFα activation (Fig. 6b,c).

In summary, this data implies that in contrast to RhoA/C, RhoB becomes upregulated and activated after TNFα stimulation. This also showed that the RhoA/B/C-WT sensors can be used as specific readouts for RhoGTPase activation.

### RhoGEFs induce RhoA/B/C activation with variable efficiencies

**Figure 7:**
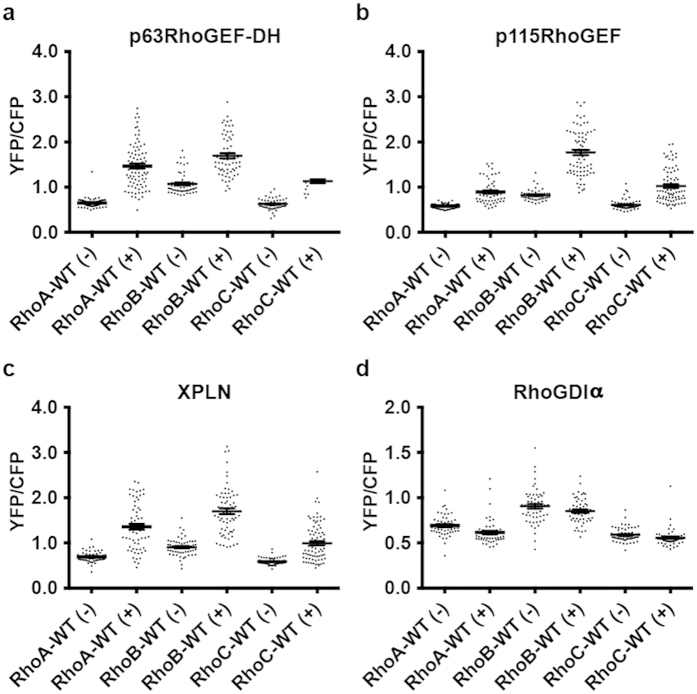
RhoGEF overexpression induces RhoA/B/C activation. **(a)** YFP/CFP ratios (±SEM) of HeLa cells transfected with the RhoA/B/C FRET sensors as indicated without (−) RhoA (n = 71), RhoB (n = 56), RhoC (n = 57) or with p63RhoGEF-DH overexpression (+) RhoA (n = 82), RhoB (n = 64), RhoC (n = 75). **(b)** YFP/CFP ratios (±SEM) of HeLa cells transfected with the RhoA/B/C FRET sensors as indicated without (−) RhoA (n = 71), RhoB (n = 43), RhoC (n = 57) or with p115RhoGEF overexpression (+) RhoA (n = 56), RhoB (n = 74), RhoC (n = 82). **(c)** YFP/CFP ratios (±SEM) of HeLa cells transfected with the RhoA/B/C FRET sensors as indicated without (−) RhoA (n = 50), RhoB (n = 56), RhoC (n = 64) or with XPLN overexpression (+) RhoA (n = 64), RhoB (n = 66), RhoC (n = 88). **(d)** YFP/CFP ratios (±SEM) of HeLa cells transfected with the RhoA/B/C FRET sensors as indicated without (−) RhoA (n = 50), RhoB (n = 56), RhoC (n = 64) or with RhoGDIα overexpression (+) RhoA (n = 53), RhoB (n = 53), RhoC (n = 55).

To further explore specificity of the RhoA/B/C-WT FRET sensors, we studied their activation following expression of different RhoGEFs and a RhoGDI variant. Sensor-expressing HeLa cells were co-transfected with either N1-mCherry as a control, mCherry-p63RhoGEF-DH (catalytic domain of p63RhoGEF), mCherry-p115RhoGEF, myc-tagged-XPLN or mCherry-RhoGDIα^30^,^40^,^47^,^48^ and YFP/CFP FRET sensor ratios were obtained as a measure of RhoGTPase activation. The RhoA/B/C-WT FRET sensors all showed responsiveness to GEF overexpression (p63RhoGEF-DH, p115RhoGEF, XPLN), although with variable efficiencies (Fig. 7a–c). Comparing control- and p63RhoGEF-DH overexpressed RhoA-WT FRET sensor cells, an increase of 225% in YFP/CFP ratio was observed, as opposed to a 180% increase for the RhoC-WT and a 158% increase for the RhoB-WT FRET sensor (Fig. 7a, Supplemental Fig. S8a). Applying the same strategy for two additional GEFs revealed the highest GEF efficiency of p115RhoGEF towards RhoB (RhoB = 215%, RhoC = 168%, RhoA = 154%) and of XPLN towards RhoA (RhoA = 196%, RhoB = 188%, RhoC = 168%) (Fig. 7b,c, Supplemental Fig. S8b,c). In contrast, minor effects were observed following expression of RhoGDIα; a small inactivation was detected for the three RhoA/B/C-WT FRET sensors upon RhoGDIα overexpression (Fig. 7d).

In conclusion, the RhoA/B/C-WT FRET sensors show differential activation by RhoGEFs, while RhoGDIα marginally alters RhoA/B/C-WT FRET sensor activation.

### RhoA/B/C FRET sensors can be used as RhoGAP readout

**Figure 8:**
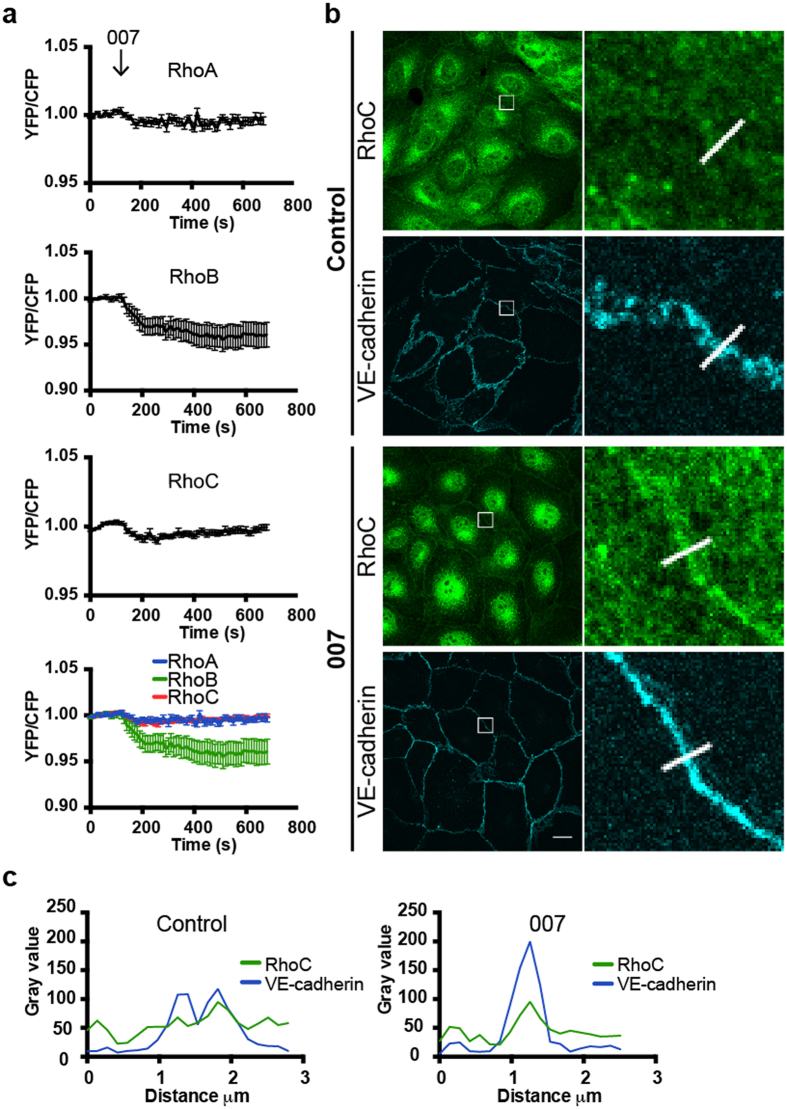
The cyclic AMP-analogue 007 induces RhoA/B/C inactivation and accumulation of RhoC at cell-cell contacts. (**a**) Normalized mean YFP/CFP ratios (±SEM) of EC that were transfected with the RhoA (n = 11), RhoB (n = 14) or RhoC (n = 10) FRET sensors and stimulated with 007 (1 mM) at t = 110 sec. (**b**) Untreated and 007-treated (1 mM, 30 minutes) EC were stained for RhoC and VE-cadherin. Box selections in control cells show colocalization of RhoC and VE-cadherin and less stabilized VE-cadherin. Boxes in 007-treated cells show co-localization of RhoC and VE-cadherin, accumulation of RhoC at cell-cell contacts and more stabilized VE-cadherin. Scale bar = 14 μm. (**c**) Profile plots show gray values of RhoC (green line) and VE-cadherin (blue line) of untreated and 007-treated EC according to the line present in the corresponding images in (**b**).

In the previous experiments, the RhoGTPase FRET sensors were used to detect GEF activity towards the different RhoGTPases. In order to investigate if these sensors can also be used to detect GAP activity, we stimulated sensor-expressing EC with a cAMP analogue (007) that is known to inactivate RhoA via Rap1, Rasip/Radil1 and ArhGAP29^49^. A small, but consistent decrease in the YFP/CFP ratio was observed in the RhoA/B/C-WT sensor-expressing cells after stimulation with 007, where RhoB inactivation was most efficient (Fig. 8a).

As a result of activating the Rap1-ArhGAP29 pathway which drives Rho inactivation, endothelial cell-cell contacts become more stabilized, reflected by an increase in transendothelial electrical resistance^49^,^50^. These findings are in line with immunofluorescence experiments that showed stable VE-cadherin complexes upon activation of this pathway (Fig. 8b). Comparing endogenous RhoC localization within endothelial monolayers revealed a clear colocalization of endogenous RhoC with VE-cadherin in 007-stimulated and unstimulated monolayers (Fig. 8b,c). However, in 007-treated cells, RhoC accumulated more prominently at cell-cell contacts.

Thus, in addition to detecting Rho activation, the RhoA/B/C-WT sensors can be deactivated, thereby serving as RhoGAP sensors. We show here that cyclicAMP-Epac1-Rap1-induced ArhGAP29 activation inhibits RhoC, which is accompanied by an increase in endogenous junctional RhoC and a more stabilized endothelial monolayer.

## Discussion

In this study we show that, in addition to the well-studied RhoGTPase RhoA, also the closely related GTPases RhoB and RhoC are activated in endothelial-cells following stimulation with GPCR-agonists as well as, for RhoB, the inflammatory cytokine TNFα. So far, it was not possible to study the activation and inactivation of RhoB and RhoC in endothelial cells with high spatio-temporal resolution. The RhoB and RhoC FRET-based sensors, described in this work, were derived from an existing RhoA FRET sensor. We show that these sensors allow visualization of the activation of these RhoGTPases in primary human EC under conditions of agonist-induced loss and gain of endothelial cell-cell contact as well as following exposure to inflammatory cytokines. These RhoA/B/C FRET sensors can readily detect GEF/GAP activity and reveal stimulus- and location-specific differences between RhoA/C and RhoB. To our knowledge, this is the first report describing spatially different activation of such highly homologous RhoGTPases in parallel in primary human cells. It is important to underscore that these sensors report GEF activity and that many RhoGEFs are known to be redundant in their GTPase activation. Although we did find preferential activation of RhoA or RhoB by selected RhoGEFs (Fig. 7), all RhoGTPases were activated by the three GEFs tested. Our results show that it is the GTPase that is encoded by the FRET sensor which plays a key role in revealing where the GEF-mediated activition occurs, further demonstrating the GTPase-specificity of this sensor approach.

Previously, studies aimed at RhoGTPase signaling in EC have mainly focused on RhoA, Rac1 and Cdc42, related GTPases that all perform unique functions in the process of cell motility^32^. Our data suggest that, in addition to these GTPases, RhoC may also be important in EC based on its high mRNA expression level. RhoC shares 88% homology with RhoA and RhoB^51^ and has recently been linked to vascular homeostasis in EC^52^. Most of the difference in primary sequence between RhoA, RhoB and RhoC exists in the hypervariable C-terminal domain, a region comprising 13–16 amino acids which is post-translationally modified by truncation, carboxymethylation and lipidation. This portion of the RhoGTPases critically determines the subcellular location of these proteins and is important for activation and downstream signaling^25^. Data on differential localization of endogenous RhoA/B/C proteins are limited due to a lack of specific antibodies for immunofluorescence studies. We here show localization of endogenous RhoC in both the cytoplasm as well as at cell-cell contacts as detected using a specific monoclonal antibody. These data show that the pool of RhoC which is present at cell-cell contacts, is highly dynamic, since the junctional localization of RhoC is transiently lost upon stimulation with Thrombin. This loss and re-localization at RhoC-positive cell-cell junctions occurs synchronous with a loss and gain in barrier function detected by the ECIS, suggesting that RhoC is involved in controlling endothelial barrier function.

For endothelial cells, Thrombin is a well-known activator of RhoA and it is generally assumed that RhoA-activation is the central event driving Thrombin-induced loss of endothelial barrier function. Approaching this biochemically with a well-characterized RhoA activation detection method^37^,^45^, we demonstrate that besides activating RhoA, Thrombin can also efficiently activate RhoC with comparable kinetics. Together, based on the observations that the junctional RhoC pool detected by immunofluorescence as well as the activation of RhoC is controlled by Thrombin stimulation of EC, we propose that RhoC disappears from cell-cell contacts once it becomes activated by Thrombin. How this phenomenon may be regulated on a molecular level remains to be determined.

By using our validated RhoA/B/C FRET sensors we were now able to visualize RhoGTPase activation with high spatiotemporal resolution. Using Thrombin as a physiologically relevant stimulus we demonstrate that all three GTPases RhoA/B/C become activated, implying that next to RhoA, also RhoB (although expressed at low levels in resting endothelium) and RhoC are involved in Thrombin signaling in EC. Secondly, our FRET sensors reveal differentially localized activation when comparing RhoA/C and RhoB. This is evident from the experiments where we stimulated EC with Thrombin, as well as after microtubule (MT) destabilization with nocodazole, which is known to activate RhoA following the release of MT-associated GEFH1^44^,^45^. It is likely that the observed differences in localized activation are the consequence of the differential targeting of the GTPases encoded by the respective FRET sensors. For example, the spatial activation profile of the RhoB sensor is likely the consequence of its vesicular localization, a feature which is not shared by RhoA/C. Additionally, while nocodazole cq. MT disruption has been exclusively linked to GEFH1 signaling towards RhoA, multiple GEFs are involved in Thrombin signaling, including GEFH1, LARG, PDZ-RhoGEF and p115RhoGEF^53^,^54^. This implies that differences in Thrombin-induced, spatial patterns of RhoGTPase activation can be explained by differentially localized GEFs that may preferentially activate either RhoA/C or RhoB.

Related findings regarding differences in spatio-temporal activation of RhoA and RhoC in rat mammary carcinoma cell or murine fibroblasts have been reported earlier^55^. In this work, a RhoC sensor encoding the Rho-binding domain of ROCK1 was used^56^. This sensor revealed peripheral RhoC activity in response to EGF, albeit that the relative increase in FRET ratios on a per-cell basis was higher in the PKN-based sensors used in the present study. Moreover, the ROCK based RhoC-WT sensor, when analyzed in cell lysates was as active as the RhoC-Q63L constitutively active mutant^56^. This is in contrast to the PKN-based sensors used in this study, which showed a 6–8 fold increase in FRET ratio of the RhoA/B/C constitutively active sensors as compared to the cognate WT versions (Fig. 3b). Whether this is due to differences in cell type (rat mammary carcinoma^55^ versus primary human endothelial cells (this study)) or the effector proteins (ROCK vs PKN) used in these sensors is presently unknown. Alternatively, it may be that expression of these sensors in endothelial cells is much lower compared to that in the HEK293 expression used previously^55^,^56^. The consequence could be that in endothelial cells, RhoGDI binding capacity is not limiting which will keep the wild-type sensors in an inactive, GDI-bound conformation.

Next to the localization-specific activation of RhoB, we also observed a higher dynamic range for the RhoB sensor compared to the RhoA/C FRET sensor. Since we excluded that this phenomenon is due to differences in expression level, we propose that this feature of the RhoB sensor is due to the relatively high affinity of the RhoB GTPase towards the PKN effector domain as compared to RhoA and RhoC. This increased affinity was previously quantified by applying proximity assays using PKN and the RhoA/B/C GTPases^57^.

In terms of sensitivity, the FRET sensors are suitable to discriminate stimulus-specific effects regarding the different RhoGTPases. It is obvious from our work and that of others, that agonists for G-protein-coupled receptors are particularly effective in fast and transient activation of RhoA (reviewed in^58^). However, in addition to the acute, Rho-mediated loss of barrier function induced by Thrombin or histamine, inflammatory cytokines as well as growth factors are also known to induce loss of endothelial cell-cell contacts. We tested activation of Rho GTPases using our new FRET sensors in response to VEGF, TGFβ and TNFα but found no fast activation. Overnight treatment of EC with TNFα is known to signal towards RhoB, but not to RhoA^46^. Combining RhoA/B/C protein expression analyses with FRET sensor experiments, we demonstrate that after 16 hours of TNFα stimulation, this cytokine does not only increase RhoB protein levels, but also specifically activates RhoB. It is known that stimulation of EC with TNFα results in an increase of RhoB protein expression, since the TNFα pathway interferes with the high level of degradation of RhoB^46^. Our finding that, in addition, the RhoB FRET-sensor shows increased activity following TNFα stimulation indicates that RhoB-specific GEF activity is upregulated in response to TNFα.

As is already accepted in the field, RhoGTPases cycle between an active, GEF-induced and inactive, GAP-promoted state (reviewed in^26^). Our RhoGTPase FRET sensors shows reversible activation, and can thus be used both as GEF- as well as GAP-activity readouts, as deduced from the activation and inactivation of the FRET sensors upon Thrombin stimulation. The FRET sensors not only report GEF activity, but can also monitor GEF efficiencies towards the different RhoGTPases since we observed variable response patterns for p63RhoGEF-DH, p115RhoGEF and XPLN respectively. Although XPLN has been described as specific RhoGEF for RhoA en RhoB, our FRET sensor approach showed that XPLN can also activate RhoC.

Stimulation of a pathway, mediated by the cAMP-regulated Rap1-GEF Epac1 using the cAMP analog 007^59^, results in activation of ArhGAP29^49^ and drives RhoA/B/C inactivation. This inactivation underscores the regulatory capacity of RhoGAPs towards the FRET sensors. The limited loss in FRET signal for RhoA/B/C, resulting from 007-mediated activation of ArhGAP29 might be because in resting EC, these FRET sensors are mostly present in their inactive conformation. This is also in line with the RhoGDIα-induced limited loss in FRET sensor activation (although for RhoB this phenomenon can also be explained by its lack of binding to RhoGDIα^25^). Thus, a further inactivation/reduction in FRET is consequently limited. This hypothesis is in line with the observation that the YFP-CFP ratios of the WT-FRET sensors are close to the values we obtain for the NB-FRET constructs, which we assume to be exclusively present in the GDP-bound, inactive conformation. In addition, this also suggests that Rho activation is not upregulated by simply overexpressing the FRET sensors in EC. While the FRET sensor approach in the context of ArhGAP29 signaling is limiting, stimulation of this GAP reveals new insights regarding the localization of RhoC by combining this approach with immunofluorescence. We demonstrate that together with inducing more stable VE-cadherin, RhoC accumulates at cell-cell contacts upon 007-mediated ArhGAP29 stimulation. Based on these observations, we propose that the RhoC pool which is present at cell-cell contacts, is inactive.

In conclusion, our study shows that the highly homologous RhoGTPases RhoA, RhoB and RhoC are important players in the context of endothelial signaling. By introducing RhoB and RhoC FRET sensors, based on a previously developed RhoA FRET sensor, we could now study activation of these RhoGTPases in increasing spatio-temporal detail, in response to various types of agonists. These sensors can readily detect both GEF as well as GAP activity, and will be valuable to further uncover the spatial (in)activation differences between RhoA/C and RhoB. Finally, the effective use of highly homologous FRET-sensors to discriminate local signaling in primary human cells, will allow comparable studies to detect differential (in)activation of homologous GTPases, such as of the Rap and Ras families.

## Materials and Methods

### Cloning strategy RhoB/C FRET sensors

Full length DNA sequences of either RhoB-WT, RhoB-G14V, RhoC-WT, RhoC-G14V or RhoC-Q63L were used for PCR (polymerase chain reaction) amplification, using forward primer 5′-GAGATCGCTAGCGCGGCCATCCGCAAGAAG-3′ and reverse primer 5′-GAGATCAAGCTTCCTGCAGGTCATAGCACCTTGCAGCAGTTG-3′ for both RhoB constructs, forward primer 5′-GAGATCGCTAGCGCTGCCATCCGGAAGAAAC-3′ and reverse primer 5′-GAGATCAAGCTTCCTGCAGGTTAGAGAATGGGACAGCCCCTC-3′ for RhoC-WT and forward primer 5′-GAGATCGCTAGCGCTGCAATCCGAAAGAAGC-3′ and reverse primer 5′- GAGATCAAGCTTCCTGCAGGTTAGAGAATGGGACAGCCCCTC -3′ for RhoC-G14V and RhoC-Q63L. Restriction sites are underlined in the primer sequences. PCR fragments were ligated into the pTriex vector backbone of the RhoA FRET sensor^39^,^40^ after cutting the PCR fragments and the vector with NheI and HindIII restriction enzymes. According to the same strategy RhoB-WT and RhoC-WT PCR fragments were also ligated into the non-binding (PKN-L59Q) pTriex RhoA FRET sensor backbone.

### Human umbilical vein endothelial cell (HUVEC) cell culture and transfection

Primary HUVEC were obtained from Lonza and cultured on fibronectin-coated culture flasks in EGM-2 medium, supplemented with singlequots (Lonza, Verviers, Belgium). HUVEC at passage #4 or #5 were transfected with 2 μg cDNA using a Neon transfection system (MPK5000, Invitrogen) and a corresponding Neon transfection kit (Invotrogen). A single pulse was generated at 1300 Volt for 30 ms, reaching on average a transfection efficiency of ±60%. After microporation, cells were seeded on fibronectin-coated glass coverslips and grown to a monolayer.

### HeLa cell culture and transfection

HeLa cells were obtained from American Tissue Culture Collection (Manassas, VA, USA) and cultured in Dulbecco’s Modified Eagle Medium (DMEM) supplied with Glutamax, 10% FBS, Penicillin (100 U/ml) and Streptomycin (100 μg/ml). Cell culture medium was provided by Invitrogen (Bleiswijk, NL).

Cells were transfected on a 24 mm glass coverslip (Menzel-Gläser, Braunschweig, Germany) using 2 μg Lipofectamine, 100 μl OptiMeM (Life Technologies, Bleiswijk, NL) and a total amount of 1 μg plasmid DNA.

### mRNA RhoGTPase expression level analyses

mRNA Expression level analyses was performed on RNA isolated from primary HUVEC using qPCR as previously described^35^.

### Reagents and antibodies

Thrombin (HCT-0020) was from Haematologin Technologies, Nocodazole (M1404) was from Sigma, recombinant human TNFα was from R&D systems and 8-pCPT-2-O-Me-cAMP-AM (007) was from Tocris Bioscience. Monoclonal antibodies (mAb) Rabbit anti-RhoA and Rabbit anti-RhoC were from Cell Signaling. Polyclonal antibody (pAb) Rabbit anti-RhoB was from Santa Cruz Biotechnology. Actin-stain 555 Phalloidin was obtained from Cytoskeleton. mAb Mouse anti-VE-cadherin/CD144 AF647 was from BD Pharmingen. mAb Mouse anti-actin for immunoblotting was from Sigma. Secondary antibody Chicken anti-Rabbit labeled with Alexa488 for immunofluorescence was from Invitrogen. Secondary HRP-labeled antibodies Goat anti-Mouse and Swine anti-Rabbit for immunoblotting were purchased from Dako.

### RNA interference

HUVEC at passage #4 were transfected with either Control siRNA (sc-37007, Santa Cruz Biotechnology) or RhoC siRNA (sc-41887, Santa Cruz Biotechnology). Control siRNA consisted of a non-targeting sequence of 20–25 nucleotides. A pool of 3 different 19–25 nucleotide target-specific RhoC siRNAs were used. Transfection was performed according to manufacturer’s instructions, using medium and transfection reagent from Santa Cruz Biotechnology. HUVEC were rinsed 6 hours after transfection and incubated for a period of 72 hours before additional treatment. Knockdown efficiency was checked on Western blot.

### Rhotekin-Rho binding domain (RBD) pull down assay

The Rhotekin-RBD pull down experiment was performed according to the method described in^60^. After stimulation in EGM2, cells were lysed for 5 min on ice in lysis buffer containing 25 mM Tris-HCl pH 7.2, 150 mM NaCl, 10 mM MgCl_2_, 1% NP-40, 5% glycerol and a protease inhibitor cocktail (Roche). Lysed cells were clarified by centrifuging for 5 min at 14000 × g and incubated with bacterial produced Glutatathion S-transferase (GST)-Rhotekin-RBD beads for ≥1 hour at 4 °C. After incubation, beads were washed 5 times with lysis buffer, eluted in SDS-sample buffer and the eluate was analyzed by Western blot with anti-RhoC antibody.

### Electrical Cell-substrate Impedance System (ECIS)

As readout for endothelial integrity, ECIS (Applied BioPhysics, New York, USA) was used to measure the barrier function of HUVEC on ECIS electrode arrays (8W10E). After pre-incubation with 10 mM L-cysteine for 15 minutes at 37 °C (Sigma), electrode arrays were coated with fibronectin (10 μg/ml, 0.9% NaCl (Sigma)) for ≥1 hour at 37 °C and subsequently 150.000 cells were seeded per well. Cells were grown to confluence and the electrical resistance was measured at a frequency of 4.000 Hz at 37 °C, 5% CO_2_.

### Confocal imaging

HUVEC were grown to confluency on FN-coated glass coverslips and stimulated or transfected as described. Cells were washed with PBS (1 mM CaCl2, 0.5 mM MgCl2) prior to fixation for 5 min with 4% formaldehyde. Fixed cells were permeabilized in PBS with 0.5% Triton X-100, blocked in PBS with 0.5% Bovine serum albumin (BSA) and subsequently incubated with primary (1 hr) and secondary (30 min) antibodies diluted in 0.5% PBS-BSA. In between every step, cells were washed in PBS. Fluorescent images were obtained on a Zeiss LSM510/Meta confocal laser-scanning microscope, equipped with Zeiss/Zen 2011 software; images were recorded using a 63x oil immersion objective (NA 1.40).

### Spectral imaging RhoB/C FRET sensors

HEK293 cells were transiently transfected with RhoB/C-NB, RhoB-G14V or RhoC-Q63L FRET sensors. 16 hours after transfection, the cells were treated with trypsin and re-suspended in ice-cold phosphate buffered saline. The cell suspensions were excited at 430 nm and scanned for fluorescence emission in a fluorometer (Fluorolog, Horiba Jobin Yvon). The raw spectra were then background-subtracted using untransfected cells.

### Live HUVEC FRET measurements

Transfected HUVEC on glass coverslips were pre-stimulated when indicated and were mounted in metal Attofluor cell chambers 18 hours after microporation. Live-cell FRET imaging was performed on a Zeiss Observer Z1 microscope, equipped with a 40x oil immersion objective (NA 1.3) and a HXP 120 Volt excitation light source. A FRET filter cube with Exciter ET 436/20 and 455 DCLP dichroic mirror (Chroma, Bellows Falls, Vermont, USA) was used to excite CFP. Emission light was directed to a second dichroic mirror (510 DSCP (Chroma, Bellows Falls, Vermont, USA)) to allow simultaneous detection of CFP and YFP in a dual camera setup. Emission wavelengths of 455–510 nm were captured on a first Hamamatsu ORCA-R2 digital CCD camera via an ET 480/40 nm emission filter (Chroma, Bellows Falls, Vermont, USA). Emission wavelengths of 510 nm and higher were captured on a second Hamamatsu ORCA-R2 digital CCD camera via an ET 540/40 nm emission filter (Ludl Electronis Products, NY, USA). Images were acquired by using Zeiss/Zen 2011 software.

FRET ratio analysis was performed in ImageJ (National Institutes of Health) as previously described^61^. Additional YFP/CFP ratio graphs were bleedthrough-corrected (62%) for the CFP emission leakage into the YFP detection channel.

### Static HeLa cell FRET measurements

Transfected HeLa cells on glass coverslips were mounted in metal Attofluor cell chambers 18 hours after transfection. Live-cell FRET imaging was performed on a widefield microscope (Axiovert 200 M; Carl Zeiss GmbH), equipped with an oil-immersion objective (Plan-Neo- fluor 40×/1.30; Carl Zeiss GmbH) and a xenon arc lamp with mono-chromator (Cairn Research, Faversham, Kent, UK). Images were recorded with a cooled charged-coupled device camera (Coolsnap HQ, Roper Scientific, Tucson, AZ, USA). Samples were excited using 420 nm light (slit width 30 nm) and a 455DCLP dichroic mirror. CFP emission was directed to a BP470/30 filter, by rotating the filter wheel YFP emission was directed to a BP535/30 filter. RFP was excited with 570 nm (slit width 10) and using a 585CXR dichroic mirror. RFP emission light was directed to a BP620/60 filter. All acquisitions were background- and bleedthrough-corrected (55%).

## Additional Information

**How to cite this article**: Reinhard, N. R. *et al.* Spatiotemporal analysis of RhoA/B/C activation in primary human endothelial cells. *Sci. Rep.* **6**, 25502; doi: 10.1038/srep25502 (2016).

## Supplementary Material

Supplementary Dataset 1

Supplementary Movie S1

Supplementary Movie S2

## References

[b1] Aird W. C. Phenotypic heterogeneity of the endothelium: I. Structure, function, and mechanisms. Circ. Res. 100, 158–173 (2007). 17272818 10.1161/01.RES.0000255691.76142.4a

[b2] Aird W. C. Phenotypic heterogeneity of the endothelium: II. Representative vascular beds. Circ. Res. 100, 174–190 (2007). 17272819 10.1161/01.RES.0000255690.03436.ae

[b3] Boon R. A., Hergenreider E. & Dimmeler S. Atheroprotective mechanisms of shear stress-regulated microRNAs. Thromb. Haemost. 108, 616–620 (2012). 22955103 10.1160/TH12-07-0491

[b4] Das S. & Halushka M. K. Extracellular vesicle microRNA transfer in cardiovascular disease. Cardiovasc. Pathol. 24, 199–206 (2015). 25958013 10.1016/j.carpath.2015.04.007

[b5] Nourshargh S., Hordijk P. L. & Sixt M. Breaching multiple barriers: leukocyte motility through venular walls and the interstitium. Nat. Rev. Mol. Cell Biol. 11, 366–378 (2010). 20414258 10.1038/nrm2889

[b6] Muller W. A. Mechanisms of Leukocyte Transendothelial Migration. Annu. Rev. Pathol. Mech. Dis. 6, 323–344 (2011). 10.1146/annurev-pathol-011110-130224 PMC3628537 21073340

[b7] Vestweber D. How leukocytes cross the vascular endothelium. Nat. Publ. Gr. 15, 692–704 (2015). 10.1038/nri3908 26471775

[b8] Vestweber D., Broermann A. & Schulte D. Control of endothelial barrier function by regulating vascular endothelial-cadherin. Curr. Opin. Hematol. 17, 230–236 (2010). 20393283 10.1097/MOH.0b013e328338664b

[b9] Schaefer A. & Hordijk P. L. Cell-stiffness-induced mechanosignaling - a key driver of leukocyte transendothelial migration. J. Cell Sci. 128, 2221–2230 (2015). 26092932 10.1242/jcs.163055

[b10] Korn C. & Augustin H. G. Mechanisms of Vessel Pruning and Regression. Dev. Cell 34, 5–17 (2015). 26151903 10.1016/j.devcel.2015.06.004

[b11] van Hinsbergh V. W. M., Eringa E. C. & Daemen M. J. A. P. Neovascularization of the atherosclerotic plaque: interplay between atherosclerotic lesion, adventitia-derived microvessels and perivascular fat. Curr. Opin. Lipidol. 26, 405–411 (2015). 26241102 10.1097/MOL.0000000000000210

[b12] Dejana E. & Vestweber D. The role of VE-cadherin in vascular morphogenesis and permeability control. Prog. Mol. Biol. Transl. Sci. 116, 119–144 (2013). 23481193 10.1016/B978-0-12-394311-8.00006-6

[b13] Mehta D. & Malik A. B. Signaling mechanisms regulating endothelial permeability. Physiol. Rev. 86, 279–367 (2006). 16371600 10.1152/physrev.00012.2005

[b14] Dejana E., Orsenigo F. & Lampugnani M. G. The role of adherens junctions and VE-cadherin in the control of vascular permeability. J. Cell Sci. 121, 2115–2122 (2008). 18565824 10.1242/jcs.017897

[b15] Lampugnani M. G. *et al.* The molecular organization of endothelial cell to cell junctions: differential association of plakoglobin, beta-catenin, and alpha-catenin with vascular endothelial cadherin (VE-cadherin). J. Cell Biol. 129, 203–217 (1995). 7698986 10.1083/jcb.129.1.203 PMC2120375

[b16] Dejana E. Endothelial adherens junctions: implications in the control of vascular permeability and angiogenesis. J. Clin. Invest. 98, 1949–1953 (1996). 8903311 10.1172/JCI118997 PMC507636

[b17] Giannotta M., Trani M. & Dejana E. VE-cadherin and endothelial adherens junctions: active guardians of vascular integrity. Dev. Cell 26, 441–454 (2013). 24044891 10.1016/j.devcel.2013.08.020

[b18] Abu Taha A. & Schnittler H.-J. Dynamics between actin and the VE-cadherin/catenin complex: novel aspects of the ARP2/3 complex in regulation of endothelial junctions. Cell Adh. Migr. 8, 125–135 (2014). 24621569 10.4161/cam.28243 PMC4049858

[b19] Vandenbroucke E., Mehta D., Minshall R. & Malik A. B. Regulation of endothelial junctional permeability. Ann. N. Y. Acad. Sci. 1123, 134–145 (2008). 18375586 10.1196/annals.1420.016

[b20] Marcos-Ramiro B., García-Weber D. & Millán J. TNF-induced endothelial barrier disruption: beyond actin and Rho. Thromb. Haemost. 112, 1088–1102 (2014). 25078148 10.1160/TH14-04-0299

[b21] Vestweber D. Cadherins in tissue architecture and disease. J. Mol. Med. (Berl). 93, 5–11 (2015). 25488198 10.1007/s00109-014-1231-5

[b22] Muller W. A. Localized signals that regulate transendothelial migration. Curr. Opin. Immunol. 38, 24–29 (2016). 26584476 10.1016/j.coi.2015.10.006 PMC4715928

[b23] Jaffe A. B. & Hall A. Rho GTPases: biochemistry and biology. Annu. Rev. Cell Dev. Biol. 21, 247–269 (2005). 16212495 10.1146/annurev.cellbio.21.020604.150721

[b24] Ridley A. J. Rho GTPases and actin dynamics in membrane protrusions and vesicle trafficking. Trends Cell Biol. 16, 522–529 (2006). 16949823 10.1016/j.tcb.2006.08.006

[b25] Schaefer A., Reinhard N. R. & Hordijk P. L. Toward understanding RhoGTPase specificity: structure, function and local activation. Small GTPases 5, 1–11 (2014). 10.4161/21541248.2014.968004 PMC4601309 25483298

[b26] Cherfils J. & Zeghouf M. Regulation of small GTPases by GEFs, GAPs, and GDIs. Physiol. Rev. 93, 269–309 (2013). 23303910 10.1152/physrev.00003.2012

[b27] Bos J. L., Rehmann H. & Wittinghofer A. GEFs and GAPs: critical elements in the control of small G proteins. Cell 129, 865–877 (2007). 17540168 10.1016/j.cell.2007.05.018

[b28] Wennerberg K., Rossman K. L. & Der C. J. The Ras superfamily at a glance. J. Cell Sci. 118, 843–846 (2005). 15731001 10.1242/jcs.01660

[b29] Wittinghofer A. & Vetter I. R. Structure-function relationships of the G domain, a canonical switch motif. Annu. Rev. Biochem. 80, 943–971 (2011). 21675921 10.1146/annurev-biochem-062708-134043

[b30] Garcia-Mata R., Boulter E. & Burridge K. The ‘invisible hand’: regulation of RHO GTPases by RHOGDIs. Nat. Rev. Mol. Cell Biol. 12, 493–504 (2011). 21779026 10.1038/nrm3153 PMC3260518

[b31] Ridley A. J. *et al.* Cell migration: integrating signals from front to back. Science 302, 1704–1709 (2003). 14657486 10.1126/science.1092053

[b32] Ridley A. J. Rho GTPase signalling in cell migration. Curr. Opin. Cell Biol. 36, 103–112 (2015). 26363959 10.1016/j.ceb.2015.08.005 PMC4728192

[b33] Beckers C. M. L., Hinsbergh V. W. M. Van & van Nieuw Amerongen G. P. Driving Rho GTPase activity in endothelial cells regulates barrier integrity. Thromb. Haemost. 103, 40–55 (2010). 20062930 10.1160/TH09-06-0403

[b34] Huveneers S., Daemen M. J. A. P. & Hordijk P. L. Between Rho(k) and a hard place: the relation between vessel wall stiffness, endothelial contractility, and cardiovascular disease. Circ. Res. 116, 895–908 (2015). 25722443 10.1161/CIRCRESAHA.116.305720

[b35] van Helden S. F. G., Anthony E. C., Dee R. & Hordijk P. L. Rho GTPase expression in human myeloid cells. PLos One 7, e42563 (2012). 22916134 10.1371/journal.pone.0042563 PMC3420873

[b36] Reid T. *et al.* Rhotekin, a new putative target for Rho bearing homology to a serine/threonine kinase, PKN, and rhophilin in the rho-binding domain. J. Biol. Chem. 271, 13556–13560 (1996). 8662891 10.1074/jbc.271.23.13556

[b37] Amerongen G. P. v. N., Delft S. v., Vermeer M. A., Collard J. G. & van Hinsbergh V. W. M. Activation of RhoA by Thrombin in Endothelial Hyperpermeability : Role of Rho Kinase and Protein Tyrosine Kinases. Circ. Res. 87, 335–340 (2000). 10948069 10.1161/01.res.87.4.335

[b38] Bogatcheva N. V., Garcia J. G. N. & Verin A. D. Molecular mechanisms of thrombin-induced endothelial cell permeability. Biochem. (Mosc). 67, 75–84 (2002). 10.1023/a:1013904231324 11841342

[b39] Lin B., Yin T., Wu Y. I., Inoue T. & Levchenko A. Interplay between chemotaxis and contact inhibition of locomotion determines exploratory cell migration. Nat. Commun. 6, 7619 (2015). 25851023 10.1038/ncomms7619 PMC4391292

[b40] van Unen J. *et al.* Plasma membrane restricted RhoGEF activity is sufficient for RhoA-mediated actin polymerization. Sci. Rep. 5, 14693 (2015). 26435194 10.1038/srep14693 PMC4592971

[b41] Lam B. D. & Hordijk P. L. The Rac1 hypervariable region in targeting and signaling: a tail of many stories. Small GTPases 4, 78–89 (2013). 23354415 10.4161/sgtp.23310 PMC3747260

[b42] Michaelson D. *et al.* Differential localization of Rho GTPases in live cells: regulation by hypervariable regions and RhoGDI binding. J. Cell Biol. 152, 111–126 (2001). 11149925 10.1083/jcb.152.1.111 PMC2193662

[b43] Fernandez-Borja M., Janssen L., Verwoerd D., Hordijk P. & Neefjes J. RhoB regulates endosome transport by promoting actin assembly on endosomal membranes through Dia1. J. Cell Sci. 118, 2661–2670 (2005). 15944396 10.1242/jcs.02384

[b44] Chang Y.-C., Nalbant P., Birkenfeld J., Chang Z.-F. & Bokoch G. M. GEF-H1 couples nocodazole-induced microtubule disassembly to cell contractility via RhoA. Mol. Biol. Cell 19, 2147–2153 (2008). 18287519 10.1091/mbc.E07-12-1269 PMC2366883

[b45] Ren Y., Li R., Zheng Y. & Busch H. Cloning and characterization of GEF-H1, a microtubule-associated guanine nucleotide exchange factor for Rac and Rho GTPases. J. Biol. Chem. 273, 34954–34960 (1998). 9857026 10.1074/jbc.273.52.34954

[b46] Kroon J., Tol S., van Amstel S., Elias J. A. & Fernandez-Borja M. The Small GTPase RhoB Regulates TNFα Signaling in Endothelial Cells. PLos One 8, e75031 (2013). 24086429 10.1371/journal.pone.0075031 PMC3784429

[b47] Birukova A. A. *et al.* Novel role of microtubules in thrombin-induced endothelial barrier dysfunction. FASEB J. 18, 1879–90 (2004). 15576491 10.1096/fj.04-2328com

[b48] Arthur W. T., Ellerbroek S. M., Der C. J., Burridge K. & Wennerberg K. XPLN, a guanine nucleotide exchange factor for RhoA and RhoB, but not RhoC. J. Biol. Chem. 277, 42964–72 (2002). 12221096 10.1074/jbc.M207401200

[b49] Post A. *et al.* Rasip1 mediates Rap1 regulation of Rho in endothelial barrier function through ArhGAP29. Proc. Natl. Acad. Sci. USA 110, 11427–32 (2013). 23798437 10.1073/pnas.1306595110 PMC3710801

[b50] Pannekoek W. J. *et al.* Epac1 and PDZ-GEF cooperate in Rap1 mediated endothelial junction control. Cell. Signal. 23, 2056–2064 (2011). 21840392 10.1016/j.cellsig.2011.07.022

[b51] Ridley A. J. RhoA, RhoB and RhoC have different roles in cancer cell migration. J. Microsc. 251, 242–249 (2013). 23488932 10.1111/jmi.12025

[b52] Hoeppner L. H. *et al.* RhoC maintains vascular homeostasis by regulating VEGF-induced signaling in endothelial cells. J. Cell Sci. 128, 3556–3568 (2015). 26136364 10.1242/jcs.167601 PMC4647168

[b53] Mikelis C. M. *et al.* PDZ-RhoGEF and LARG are essential for embryonic development and provide a link between thrombin and LPA receptors and Rho activation. J. Biol. Chem. 288, 12232–12243 (2013). 23467409 10.1074/jbc.M112.428599 PMC3636907

[b54] Meiri D. *et al.* Mechanistic insight into GPCR-mediated activation of the microtubule-associated RhoA exchange factor GEF-H1. Nat. Commun. 5, 4857 (2014). 25209408 10.1038/ncomms5857

[b55] Bravo-Cordero J. J. *et al.* A novel spatiotemporal RhoC activation pathway locally regulates cofilin activity at invadopodia. Curr. Biol. 21, 635–644 (2011). 21474314 10.1016/j.cub.2011.03.039 PMC3081966

[b56] Zawistowski J. S., Sabouri-Ghomi M., Danuser G., Hahn K. M. & Hodgson L. A RhoC biosensor reveals differences in the activation kinetics of RhoA and RhoC in migrating cells. PLos One 8, e79877 (2013). 24224016 10.1371/journal.pone.0079877 PMC3818223

[b57] Hutchinson C. L., Lowe P. N., McLaughlin S. H., Mott H. R. & Owen D. Differential binding of RhoA, RhoB, and RhoC to protein kinase C-related kinase (PRK) isoforms PRK1, PRK2, and PRK3: PRKs have the highest affinity for RhoB. Biochemistry 52, 7999–8011 (2013). 24128008 10.1021/bi401216w

[b58] Yu O. M. & Brown J. H. G Protein-Coupled Receptor and RhoA-Stimulated Transcriptional Responses: Links to Inflammation, Differentiation, and Cell Proliferation. Mol. Pharmacol. 88, 171–180 (2015). 25904553 10.1124/mol.115.097857 PMC4468647

[b59] Enserink J. M. *et al.* A novel Epac-specific cAMP analogue demonstrates independent regulation of Rap1 and ERK. Nat. Cell Biol. 4, 901–906 (2002). 12402047 10.1038/ncb874

[b60] Sander E. E., Ten Klooster J. P., Van Delft S., Van Der Kammen R. A. & Collard J. G. Rac downregulates Rho activity: Reciprocal balance between both GTPases determines cellular morphology and migratory behavior. J. Cell Biol. 147, 1009–1021 (1999). 10579721 10.1083/jcb.147.5.1009 PMC2169355

[b61] Timmerman I. *et al.* A local VE-cadherin and Trio-based signaling complex stabilizes endothelial junctions through Rac1. J. Cell Sci. 128, 3041–3054 (2015). 26116572 10.1242/jcs.168674

