# Supplementary material for: Spatiotemporal analysis of RhoA/B/C activation in primary human endothelial cells
Source: Sci Rep. 2016 May 5;6:25502. doi: 10.1038/srep25502 (PMC4857094; doi:10.1038/srep25502)
Supplement: Supplementary Dataset 1 [file srep25502-s1.pdf]

Supplementary materials:

Spatiotemporal analysis of RhoA/B/C activation in primary human endothelial cells

Nathalie R. Reinhard[1,2], Suzanne F. van Helden[2], Eloise C. Anthony[2], Taofei Yin[3]., Yi Wu[3], Joachim Goedhart[1], Theodorus W.J. Gadella Jr.[1], Peter L. Hordijk[1,2,* †]

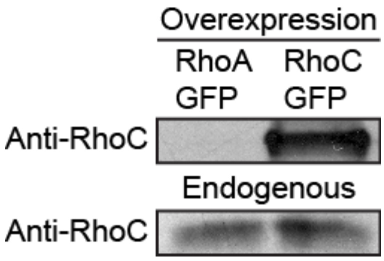

Supplemental Figure S1: RhoC antibody specifically detects RhoC and not RhoA. ECs were transfected with RhoA-GFP or RhoC-GFP and a RhoC antibody was used to check for its specifity towards RhoA and RhoC on Western blot. Endogenous RhoC detection in RhoA-GFP and RhoC-GFP positive cell lysates was included to demonstrate that endogenous RhoC was detected in both conditions.

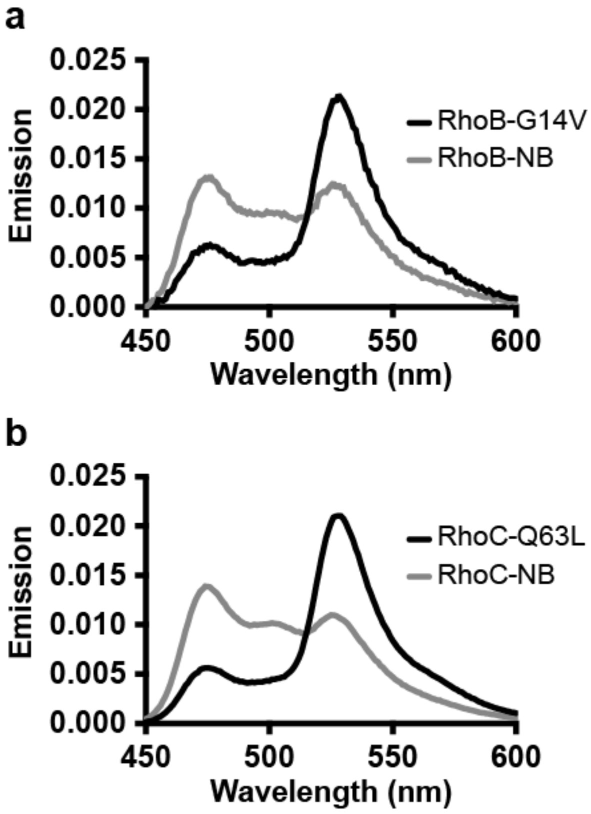

Supplemental Figure S2: Emission spectra of RhoB and RhoC FRET sensors. (**a**) Normalized emission spectra of the RhoB-G14V (black trace) and RhoB-DN (grey trace) measured in transiently transfected HEK293 cells. Spectra were normalized to the area under the curve. (**b**) Normalized emission spectra of the RhoC-Q63L (black trace) and RhoC-DN (grey trace) measured in transiently transfected HEK293 cells. Spectra were normalized to the area under the curve.

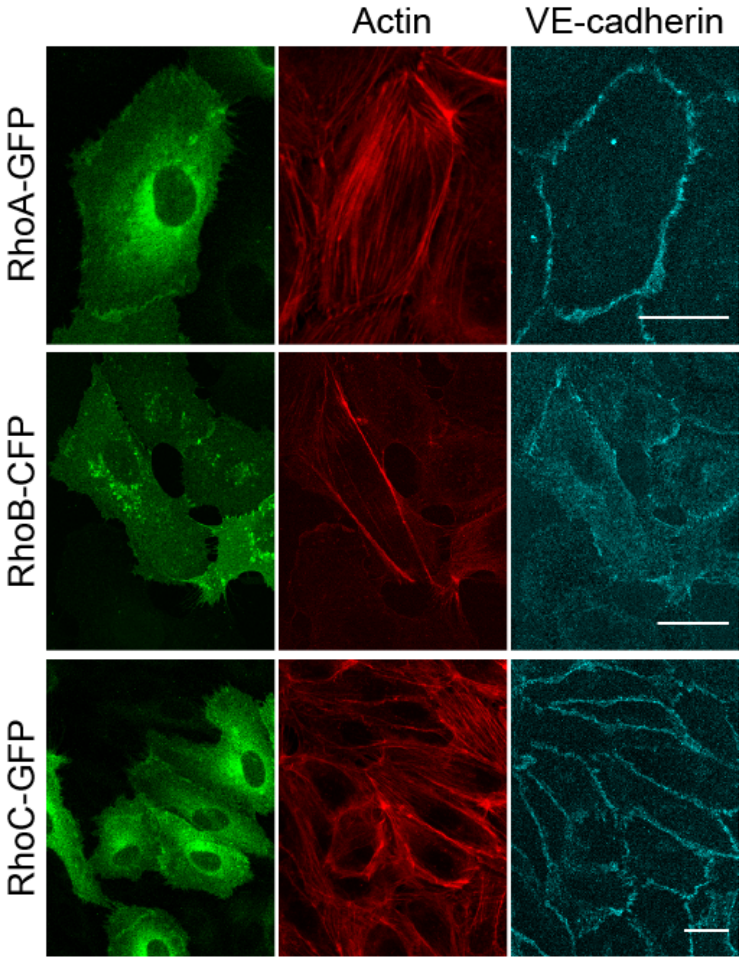

Supplemental Figure S3: Localization of FP-tagged RhoA/B/C constructs. ECs were transfected with RhoA/C-GFP or RhoB-CFP and co-stained for F-actin and VE-cadherin. Scale bar = 25µm.

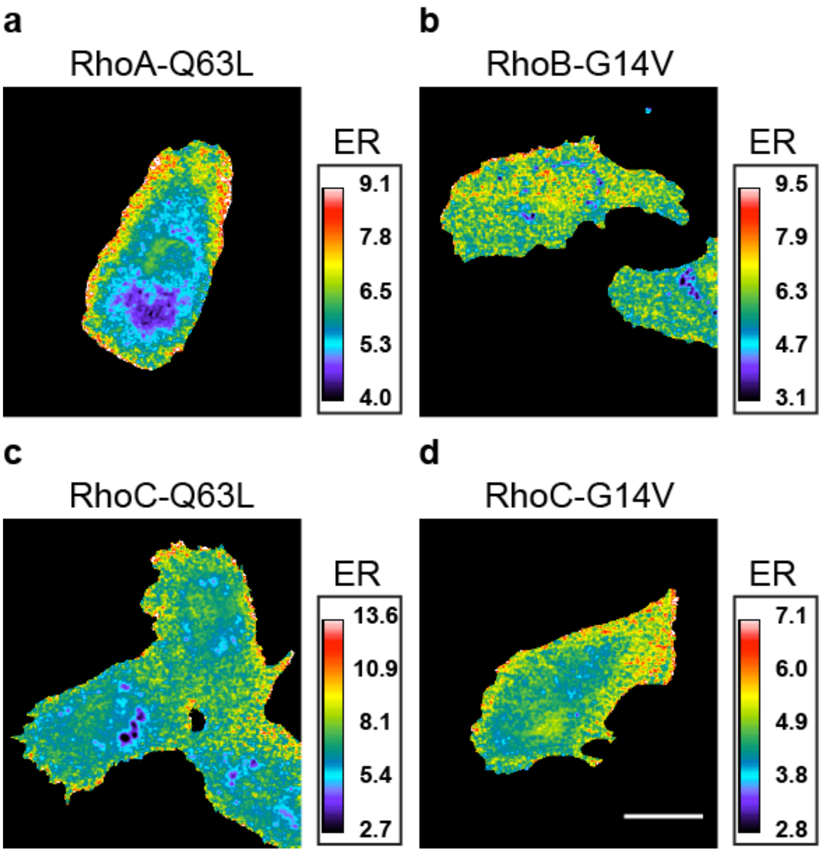

Supplemental Figure S4: Dominant positive RhoA/B/C FRET sensors. (**a-d**) Ratiometric images of ECs that were transfected with the (**a**) RhoA-Q63L- (**b**) RhoB-G14V- (**c**) RhoC-Q63L- or (**d**) RhoC-G14V FRET sensors. Warm colors represent high YFP/CFP ratios (Emission ratio (ER) on the right). Scale bar = 25µm.

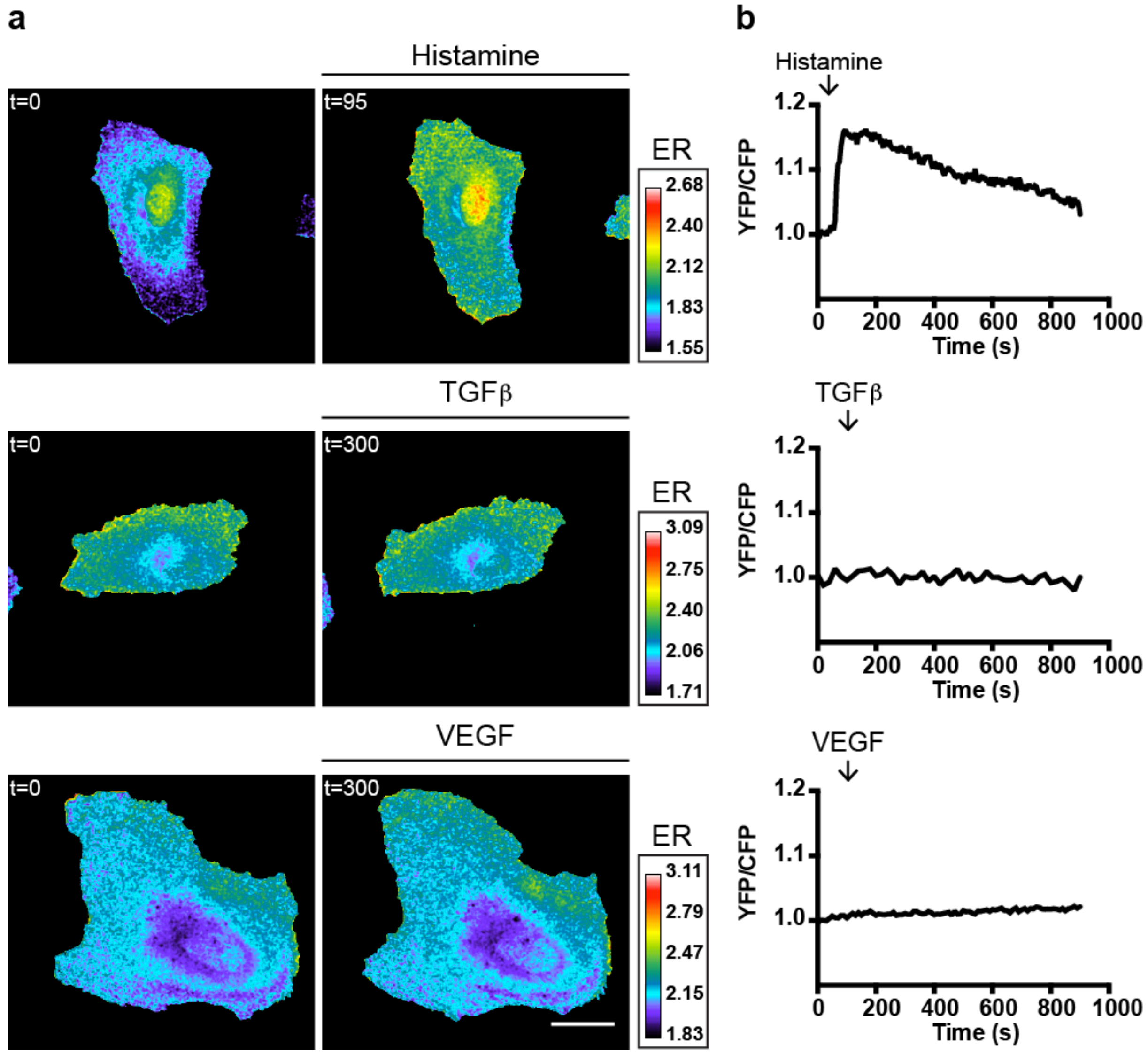

Supplemental Figure S5: Histamine, but not TGFβ or VEGF, induces RhoA activation in human ECs. (**a**) Ratiometric images of ECs that were transfected with the RhoA FRET sensor and stimulated with either Histamine (100 μM) at t=55 sec, VEGF (50ng/ml) at t=110 sec, or TGFβ (10 ng/ml) at t=100 sec. Warm colors represent high YFP/CFP ratios (Emission ratio (ER) on the right). Scale bar=25 μm. (**b**) Normalized mean YFP/CFP ratio changes of the cells in (**a**), which are representative examples of 2-7 cells.

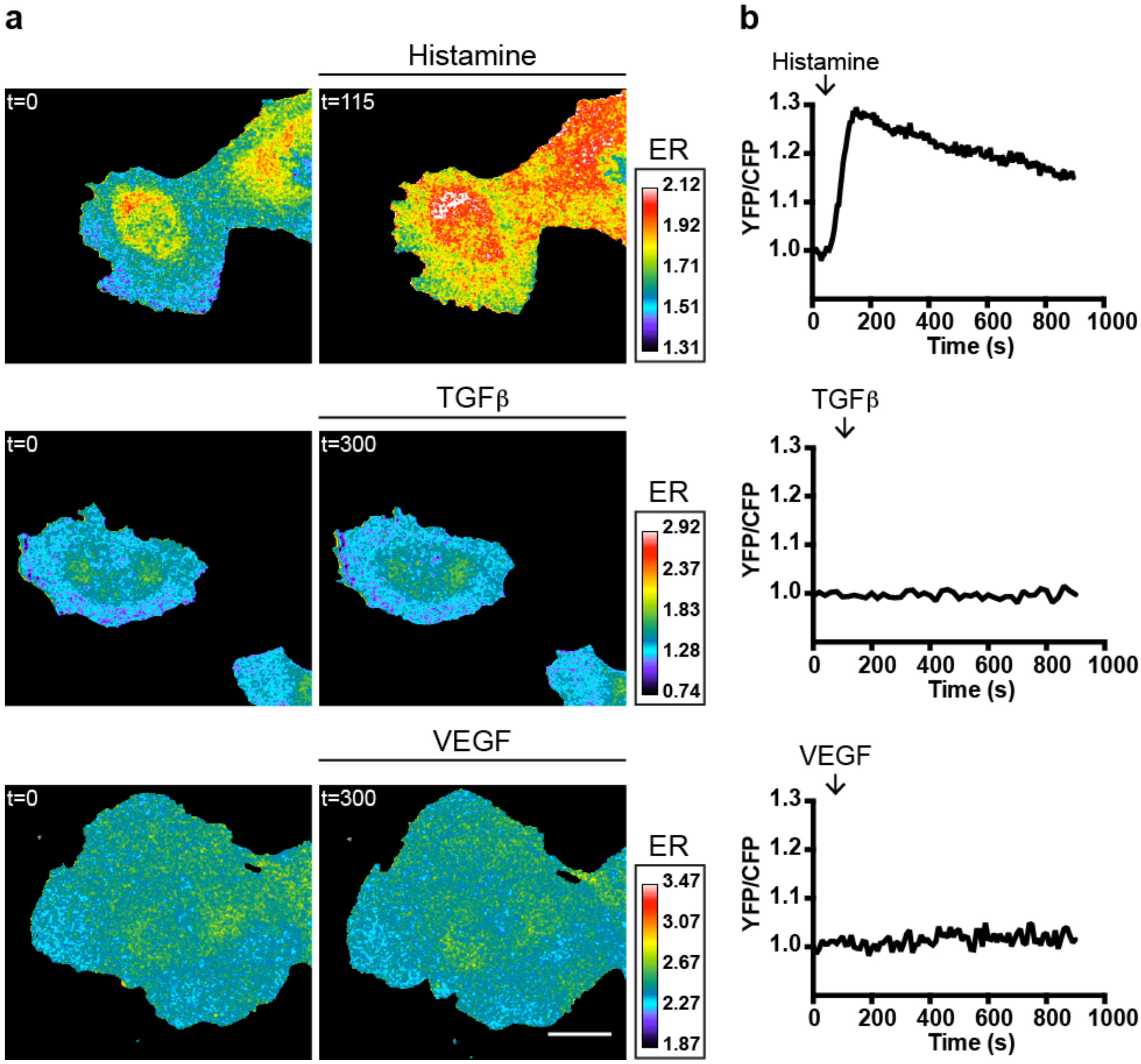

Supplemental Figure S6: Histamine, but not TGFβ or VEGF, induces RhoB activation in human ECs. (**a**) Ratiometric images of ECs that were transfected with the RhoB FRET sensor and stimulated with either Histamine (100 μM) at t=55 sec, VEGF (50ng/ml) at t=110 sec, or TGFβ (10 ng/ml) at t=100 sec. Warm colors represent high YFP/CFP ratios (Emission ratio (ER) on the right). Scale bar=25 μm. (**b**) Normalized mean YFP/CFP ratio changes of the cells in (**a**), which are representative examples of 2-7 cells.

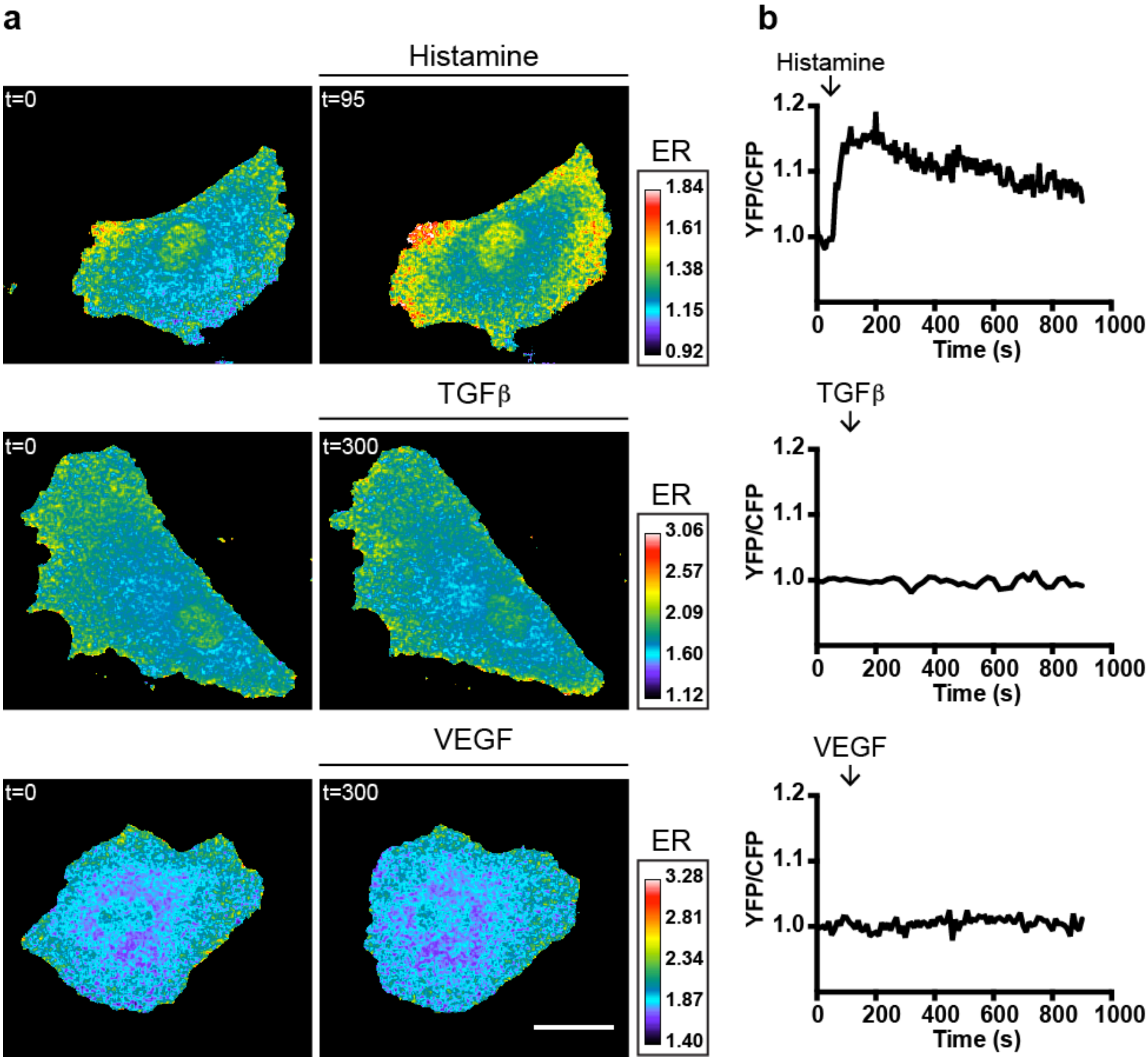

Supplemental Figure S7: Histamine, but not TGFβ or VEGF, induces RhoC activation in human ECs. (**a**) Ratiometric images of ECs that were transfected with the RhoC FRET sensor and stimulated with either Histamine (100 µM) at t=55 sec, VEGF (50ng/ml) at t=110 sec, or TGFβ (10 ng/ml) at t=100 sec. Warm colors represent high YFP/CFP ratios (Emission ratio (ER) on the right). Scale bar=25 µm. (**b**) Normalized mean YFP/CFP ratio changes of the cells in (**a**), which are representative examples of 2-7 cells.

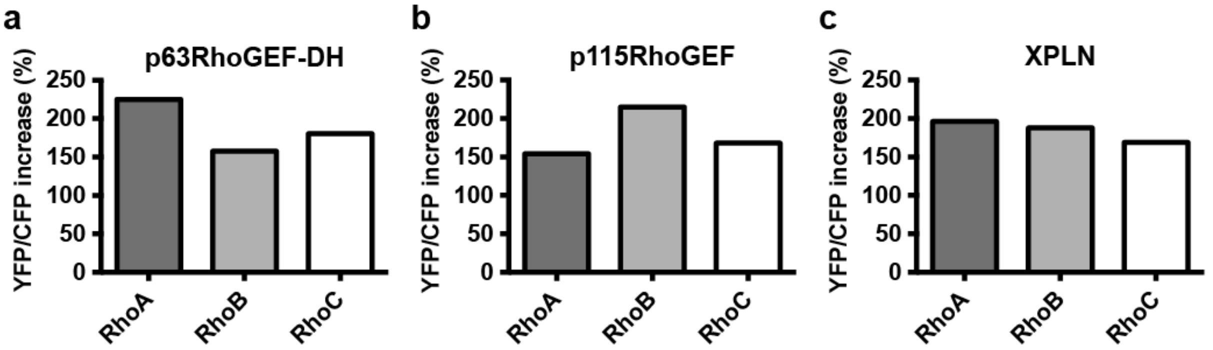

Supplemental Figure S8: RhoGEF efficiences towards RhoA/B/C. (**a,b.c**) Mean YFP/CFP ratios of RhoA/B/C FRET sensors with RhoGEF overexpression were divided by the mean YFP/CFP ratios of RhoA/B/C FRET sensors without RhoGEF overexpression to illustrate GEF efficiences of (**a**) p63RhoGEF-DH (**b**) p115RhoGEF and (**c**) XPLN towards the different RhoA/B/C-WT FRET sensors. Mean values, used for the calculation, were obtained from Fig. 7a-c.

Supplemental Movie M1:

Thrombin induces RhoA/B/C activation

Primary HUVEC transfected with the RhoA (left panel); RhoB (middle panel); RhoC (right panel); FRET sensors were stimulated with 1U/ml Thrombin at t=110 sec. See also Fig. 3.

Supplemental Movie M1:

Nocodazole induces RhoA/B/C activation

Primary HUVEC transfected with the RhoA (left panel); RhoB (middle panel); RhoC (right panel); FRET sensors were stimulated with 5 μM nocodazole to induce microtubule depolymerization at t=180 sec. See also Fig. 4.
